# Radiotherapy quality assurance in the PROTECT trial – a European randomised phase III-trial comparing proton and photon therapy in the treatment of patients with oesophageal cancer

**DOI:** 10.2340/1651-226X.2025.42774

**Published:** 2025-03-13

**Authors:** Camilla S. Byskov, Hanna R. Mortensen, Marie-Claude Biston, Sara Broggi, Rebecca Bütof, Richard Canters, Gilles Crehange, Gilles Defraene, Jerome Doyen, Mai L. Ehmsen, Silvia Fabiano, Francesco Fracchiola, Farid Goudjil, Karin Haustermans, Sarah E. Jensen, Maria F. Jensen, Marie Lecornu, Sebastian Makocki, Aurélia L. Mana, Andrea Martignano, Arturs Meijers, Alfredo Mirandola, Diana A. Mitrea, Christina T. Muijs, Ditte S. Møller, Marianne Nordsmark, Ester Orlandi, Panagiotis Balermpas, Pieter Populaire, Daniele Scartoni, Jessica Serrand, Muhammad Shamshad, Najla Slim, Valentina Vanoni, Anthony Vela, Marie Vidal, Gloria Vilches-Freixas, Damien Weber, Lone Hoffmann

**Affiliations:** a Department of Oncology, Aarhus University Hospital, Aarhus, Denmark; b Danish Centre for Particle Therapy, Aarhus University Hospital, Aarhus, Denmark; c Centre Léon Bérard, Lyon, France; d San Raffaele Hospital, Milan, Italy; e Department of Radiotherapy and Radiation Oncology, Faculty of Medicine and University Hospital Carl Gustav Carus, TUD Dresden University of Technology, Dresden, Germany; f OncoRay – National Center for Radiation Research in Oncology, Faculty of Medicine and University Hospital Carl Gustav Carus, TUD Dresden University of Technology, Helmholtz-Zentrum Dresden-Rossendorf, Dresden, Germany; g Department of Radiation Oncology (Maastro), GROW School for Oncology and Reproduction, Maastricht University Medical Centre+, Maastricht, The Netherlands; h Curie Institute, Paris, France; i KU Leuven – University of Leuven – Department of Oncology – Laboratory of Experimental Radiotherapy, Leuven, Belgium; j Centre Antoine Lacassagne, Nice, France; k University Hospital Zurich, Zurich, Switzerland; l Department of medical physics, APSS, Trento, Italy; m Department of Radiation Oncology, University Hospitals Leuven, Leuven, Belgium; n Centre François Baclesse, Caen, France; o Paul Scherrer Institute, Villigen, Switzerland; p National Centre of Oncology Adrotherapy, Pavia, Italy; q Department of Radiation Oncology, University Medical Center Groningen, University of Groningen, Groningen, The Netherlands; r Department of Clinical Medicine, Faculty of Health Sciences, Aarhus University, Aarhus, Denmark; s Department of Clinical, Surgical, Diagnostic, and Pediatric Sciences, University of Pavia, Pavia, Italy; t Clinical Department, National Center for Oncological Hadrontherapy (Fondazione CNAO), Pavia, Italy; u Department of Proton Therapy, APSS, Trento, Italy; v EBG MedAustron GmbH, Wiener Neustadt, Austria; w Department of Radiation Oncology, APSS, Trento, Italy

**Keywords:** Radiotherapy quality assurance, clinical trial, oesophageal cancer

## Abstract

**Purpose:**

To present results from the trial radiotherapy quality assurance (RTQA) programme of the centres involved in the randomised phase-III PROton versus photon Therapy for esophageal Cancer – a Trimodality strategy (PROTECT)-trial, investigating the clinical effect of proton therapy (PT) vs. photon therapy (XT) for patients with oesophageal cancer.

**Materials and methods:**

The pre-trial RTQA programme consists of benchmark target and organ at risk (OAR) delineations as well as treatment planning cases, a facility questionnaire and beam output audits. Continuous on-trial RTQA with individual case review (ICR) of the first two patients and every fifth patient at each participating site is performed. Patient-specific QA is mandatory for all patients. On-site visits are scheduled after the inclusion of the first two patients at two associated PT and XT sites. Workshops are arranged annually for all PROTECT participants.

**Results:**

Fifteen PT/XT sites are enrolled in the trial RTQA programme. Of these, eight PT/XT sites have completed the entire pre-trial RTQA programme. Three sites are actively including patients in the trial. On-trial ICR was performed for 22 patients. For the delineation of targets and OARs, six major and 11 minor variations were reported, and for six patients, there were no remarks. One major and four minor variations were reported for the treatment plans. Three site visits and two annual workshops were completed.

**Interpretation:**

A comprehensive RTQA programme was implemented for the PROTECT phase III trial. All centres adhered to guidelines for pre-trial QA. For on-trial QA, major variations were primarily seen for target delineations (< 30%), and no treatment plans required re-optimisation.

## Introduction

Radiotherapy (RT) with photon therapy (XT) is standard of care for patients with locally advanced oesophageal cancer in combination with chemotherapy followed by surgery [1]. The oesophagus is in close proximity to vital organs at risk (OAR), e.g. heart and lungs, and the treatment will often result in radiation-induced side effects [2–7]. Proton therapy (PT) may reduce the dose to the surrounding OAR, whilst target dose remains similar, hence, can potentially decrease the radiation-induced side effects [6]. The European randomised phase III PROTECT-trial (PROton versus photon Therapy for Oesophageal Cancer – a Trimodality strategy; NCT05055648) will investigate the clinical effect of PT versus standard XT for oesophageal cancer patients treated with chemoradiotherapy followed by surgery [8].

It has previously been shown that a thorough quality assurance (QA) programme is important to reduce the number of major protocol deviations and to ensure high-quality results from clinical trials [9–14]. Protocol deviations are associated with increased risk of treatment failure and overall mortality [15] and are more frequent in centres where only few patients are recruited [12, 16]. In recent clinical trials involving RT of gastric and pancreatic cancer as well as for paediatric RT, resubmissions of target delineations were needed for approximately one-third of patients [16–18]. Also, regular completion of facility questionnaires (FQs), on-site visits and beam dosimetry audits are essential parts for the completion of international multicentre clinical trials and to prevent treatment heterogeneities from invalidating the significance of trial endpoints [19–21].

Therefore, in the PROTECT-trial, strict mandatory QA guidelines have been developed [8]. The completion of a pre-trial radiotherapy quality assurance (RTQA) programme consisting of benchmark delineation and treatment planning cases is mandatory, and prospective individual case reviews (ICRs) are performed for the first two patients, followed by every fifth patient at each treatment site. As part of the QA procedure, FQs have been distributed to gain insight in the patient treatment workflow across the participating centres and to ensure that all sites can deliver RT treatment according to the study protocol. A site visit is performed for associated PT and XT sites after the inclusion of the first two patients at each site, where all parts of the treatment procedure are discussed with members of the PROTECT RTQA group. Furthermore, all sites are required to perform patient-specific (PS) QA and to have a dosimetry audit performed.

The aim of this study was to present the first results from the QA programme under the PROTECT-trial, including FQs, pre-trial and on-trial RTQA, experiences from the first site visits completed as well as the two annual RTQA workshops.

## Materials and methods

In short, the PROTECT trial aims to include 396 patients with locally advanced oesophageal cancer. Patients are randomised (1:1) to either neoadjuvant proton or photon chemoradiotherapy treatment [8]. Delineation guidelines have been published [22], and for all patients, both a PT and an XT treatment plan must be optimised using inverse planning, which should fulfil the requirements in the comprehensive RTQA document of the trial (Supplementary material S1).

**Figure 1:**
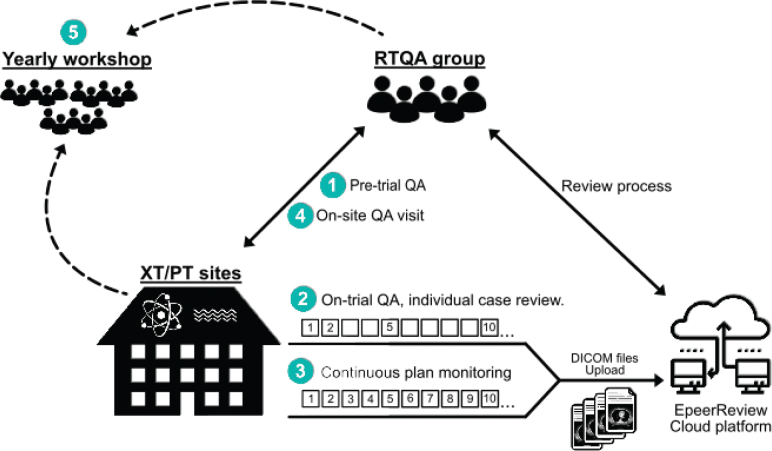
The PROTECT RTQA programme consists of (1) Pre-trial QA, (2) On-trial QA, (3) Continuous plan monitoring in ePeerReview, (4) On-site visits and (5) Annual workshops for everyone involved in the trial RTQA.

The RTQA programme consists of five elements (Figure 1):

1. Pre-trial QA:
  - Five delineation benchmark cases
  - Four treatment planning benchmark cases
  - PT/XT FQ
  - Dosimetry audit
  - A plan for handling equipment breakdown
2. On-trial QA:
  - Prospective ICR of the first two patients enrolled in the trial at each centre
  - ICR of every fifth patient
  - Secondary dose calculation for all patients
  - Patient-specific QA for all patients
3. Continuous PT/XT treatment plan monitoring performed by the RTQA group.
4. On-site QA visit:
  - Discussion on computed tomography (CT) scans, delineation, treatment planning, treatment delivery, image guidance, treatment adaption strategy and plan QA. Criteria are set based on the trial RTQA document (Supplementary material S1).
5. Annual workshop. All centres will discuss ICRs, technical solutions and scientific work.

### RTQA group

The RTQA group consists of participants from six European centres, including four radiation oncologists, seven medical physicists and two RTQA managers. The RTQA group members arrange bimonthly meetings, where issues related to the approval of benchmark cases, patient inclusion, data acquisition, ICR, data analyses, etc. are discussed.

### RTQA Programme, pre-trial QA

Five benchmark cases were prepared for pre-trial QA of both target and OAR (Body, Bowel Cavity, Heart, Liver, Lungs, Spinal Cord, planning organ at risk volume (PRV) Spinal Cord, Skin, Spleen, Kidneys and Stomach) delineations. The delineation benchmark cases were selected to represent different clinical stages with variations in location of the primary tumour and pathological lymph node(s). Target volumes and OARs were delineated according to published guidelines [22]. For target volumes, the mid-ventilation phase of the planning 4D-CT scan was used for delineation of the gross tumour volume of the primary tumour (GTVp), pathological lymph nodes (GTVn), the clinical target volume of the primary tumour (CTVp), the nodes (CTVn) and the total volume (CTVtotal). Generating an internal clinical target volume (iCTV) to account for respiratory motion was mandatory. All delineations had to be reviewed and approved by the RTQA group prior to inclusion of patients in the trial.

Four treatment planning benchmark cases were prepared and were chosen to represent different target sizes and locations for oesophageal cancer patients. The treatment planning benchmark cases are briefly described here and have been published previously [23]. When including patients in the PROTECT trial, each centre must choose between either a prescription dose of 41.4 Gy in 23 fractions or 50.4 Gy in 28 fractions. However, for the benchmark cases, prescription dose had to be 50.4 Gy in 28 fractions. Both a planning 4D-CT scan and a surveillance 4D-CT scan (recorded after 10 fractions) were included, and each centre had to calculate plan robustness towards setup and range uncertainties as well as respiratory motion. The impact of anatomical changes was evaluated using the surveillance scan. The plans were reviewed by the RTQA group, and feedback was given on primary and secondary dose constraints along with a report on mean doses to the heart and lungs compared to the results from the first eight European centres [23]. All benchmark plans had to be reviewed and approved by the RTQA group prior to inclusion of patients in the trial.

For both the delineation and the treatment planning benchmark cases, centres were asked to comply with the PROTECT RTQA guidelines (Supplementary file S1), and if these were not met, revisions had to be made, and delineations and plans re-submitted.

### Facility questionnaires

The FQs contain questions related to all parts of the treatment procedure, including pre-treatment imaging, contouring, treatment planning and delivery, treatment interruptions and dose verification for both treatment modalities. The questionnaires are updated and collected yearly, and participants are asked to submit a new FQ if treatment procedures are changed during the study period.

### RTQA programme, on-trial QA

For the first two patients included at each site, an accelerated QA programme must be performed. Target and OAR delineations as well as treatment plans must be reviewed within 24 hours from upload and a minimum of one working day before the patient will receive the first RT treatment. Delineations and treatment plans must adhere to the PROTECT RTQA guidelines (Supplementary material S1), which are also described by Thomas et al. [22] and Hoffmann et al. [23]. If either the delineations or the treatment plans do not adhere to the guideline requirements, corrections must be made as soon as possible, and treatment plans must be re-optimised within three fractions. A major protocol variation regarding target definition is defined as any case with the risk of violating target coverage throughout the treatment course. This includes but is not restricted to:

- Failure to generate an iCTVtotal
- Not including the entire oesophagus, where the CTV includes the GTV
- GTV not being within 10 mm of the propagation described on diagnostic CT, positron emission tomography (PET)-CT or endoscopic ultrasound will be discussed with the treating physician. If no substantial clinical reason, the GTV should be corrected. Smaller GTV will be considered a major variation and larger GTV a minor variation
- Less than 10 mm margin to metastatic lymph nodes unless shaping towards bone, vessels, etc.
- Not adding the described margins
- Scan range not sufficient according to section 1.1
- Not delineating all OARs included in the scan according to section 1.1 or not delineating OARs in the whole organ extent.

Violation of a first priority constraint (Table 1) for the nominal treatment plan is considered a major protocol variation except if this is deemed clinically necessary. Minor variations are defined as all other deviations from consensus guidelines due to clinical decision.

**Table 1:** Mandatory first priority constraints of target and organs at risk.

**First priority constraints, target**		
**Target**	**Dose (nominal plan)**	**Dose (all scenarios)**
iCTVtotal	V95%_iCTVtotal_ >99%	V95%_iCTVtotal_ >97%
iCTVtotal	98% < Dmean < 102%	
**First priority constraints, OAR**		
**Organs at risk**	**Dose (nominal plan)**	**Dose (all scenarios)**
Spinal Cord	D_0.05cm3_ < 45Gy	D_0.05cm3_ < 50Gy
Spinal Cord PRV	D_0.05cm3_ < 50Gy	
Lungs	MLD < 20Gy V_20Gy_ < 35% V_5Gy_ < 70%	Not evaluated
Body	D_0.05cm3_ < 110%	D_1cm3_ < 110%
	D_1cm3_ < 107%	D_5cm3_ < 107%

A major variation is noted if these are not fulfilled unless deemed clinically necessary. OAR: organs at risk.

After the first two case reviews, every fifth patient must be reviewed within a week from the treatment start. A delineation and a treatment planning checklist have been developed to ensure consistency when the members of the RTQA group perform the reviews (Supplementary materials S2 and S3). For the patients who are not part of the on-trial QA, delineations, treatment plans and surveillance, CT scans as well as re-plans must be uploaded giving the RTQA group access to monitor on-trial data continuously. All on-trial data are uploaded to the ePeerReview Cloud platform (Varian medical Systems).

### Image-guidance and adaptation

Image guidance based on volumetric imaging (enabling soft tissue visualisation and registration) should be performed daily before treatment and should be used for matching to ensure high precision of the target position during each treatment fraction. For XT, a soft tissue match on the target is preferred for daily setup. For PT, a bone match or soft tissue match on the target should be used [24]. Fiducial markers applicable for PT may be used but are not mandatory [25, 26].

The images acquired for daily image guidance must be used to verify the inter-fractional reproducibility of the target positioning. Each department must have a treatment adaptation strategy. The strategy and tolerance limits for target and OARs should be based on margins or uncertainties as determined by the individual centre. In case the tolerance limits are exceeded, action based on the adaptive strategy of the treating XT or PT centre must be performed. Adaptation should be performed within two working days.

### Continuous PT/XT treatment plan monitoring

For all patients, both a PT and an XT treatment plan are inversely planned. Both PT and XT treatment plans for all patients must be uploaded in a pseudonymised form to the central dose plan bank, where the plans will be continuously monitored for adherence to the PT/XT guidelines. In case of adaptation, the recalculated plan and dose cube based on the adaptive plan for the modality used for treatment should also be exported. No strict time demand is set for continuous monitoring. Monitoring of plans will be used to check for variations of constraints and used for feedback to the centres. Furthermore, the continuous QA programme will monitor the difference in dose to OARs in the two arms.

### Patient-specific QA

Patient-specific QA is mandatory, and all treatment plans should pass QA procedures with a γ (5%, 3 mm) above 95% or γ (3%, 3 mm) above 90%. The γ evaluation must be based on measurements of the treatment plans at the accelerator and may be based on the PSQA device used by the individual treatment site. All treatment plans must pass evaluation in a secondary, independent dose calculator with γ (5%, 3 mm) above 95% or γ (3%, 3 mm) above 90% [27].

### Site visits

When two patients have been included for both PT and at least one associated XT site, a site visit must be performed. A site visit checklist is filled out prior to the visit for both the PT and the XT sites (Supplementary materials S4a b). At the site visit, a radiation oncologist, a physicist and a dosimetrist from the site to be audited and a minimum of two participants (a physician and a physicist) from the PROTECT RTQA group must be present. All parts of the treatment of oesophageal cancer patients are discussed. A site visit report is written, and the RTQA group will ask the site to change practice if needed (Supplementary material S5).

### Dosimetry audit

It is mandatory for all sites included in the trial to have a dosimetry audit performed within the timeframe of the project. The audit should adhere to the European Organisation for Research and Treatment of Cancer (EORTC) guidelines [28]. Sites that do not have a dosimetry audit performed within 2 years of enrolment in the RTQA programme have the possibility to participate in an audit performed by IBA (IBA Dosimetry GmbH, Schwarzenbruck, Germany). The IBA audits are based on ion chamber measurements and measure absolute dose in the reference point for XT. For PT measurements, three monoenergetic fields (100, 170 and 220MeV) with a measurement depth of 2 cm and three modulated spread-out Bragg peaks (SOBP) measured in midpoint of SOBP were selected according to the IAEA TRS-398 Rev. 1 code of practice.

### Annual workshop

An online workshop is arranged yearly by the RTQA group, where participants from all activated sites are invited. Here, the status of the trial is presented by the RTQA group, and topics on, e.g., adaptive strategies, challenging cases and translational studies can be discussed.

## Results

Pre-trial QA: Eight PT sites and eight corresponding XT sites have completed the entire RTQA pre-trial programme and are approved for inclusion of patients (Table 2). Two Dutch centres also completed the pre-trial RTQA programme but will not include patients in the trial since in The Netherlands, a model-based approach is used for patient selection for PT. All sites received feedback upon completion of the benchmark cases and re-submitted delineations and plans if needed until everything was approved. Ten sites have started but not yet completed delineation and treatment planning benchmark cases.

**Table 2:** Overview of trial RTQA status.

Country	Centre	Pre-trial RTQA				On-trial RTQA	
		Benchmark delineations	Benchmark treatment plans	Facility Questionnaire	Beam Output Audit	Individual case reviews	Patients included
Belgium	# 1 (PT+XT)	OK	OK	OK	OK	PT: 5	30
						XT: 5	
Denmark	# 2 (PT+XT)	OK	OK	OK	OK	PT: 5	31
						XT: 4	
Switzerland	# 3a (PT)	OK	OK	OK	OK	2	2
	# 3b (XT)	OK	OK	OK	OK	1	1
Germany	# 4 (PT+XT)	OK	OK	OK	OK		
Italy	# 5a (PT)	OK	OK	OK	OK		
	# 5b (XT)	OK	OK	OK	OK		
	# 6a (PT)	OK	OK	OK	OK		
	# 6b (XT)	OK	OK	OK	OK		
France	# 7 (PT+XT)	OK	OK	OK	OK		
	# 8 (PT+XT)	OK		OK	PT: OK		
					XT		
	# 9 (PT plans + XT)	OK	OK	OK	OK		
	# 10 (PT+XT)		OK	OK	OK		
Norway	# 11 (PT+XT)						
	# 12 (PT+XT)						
Germany	# 13 (PT+XT)						
The Netherlands	# 14 (PT+XT)	OK	OK	OK	OK	Dutch model-based approach	
	# 15 (PT+XT)	OK	OK	OK	OK	Dutch model-based approach	

XT: Photon therapy; PT: proton therapy; RTQA: radiotherapy quality assurance. The Netherlands have finished the pre-trial RTQA programme but will not include patients in the PROTECT trial since they select patients for PT with a model-based selection.

FQs were distributed to 28 sites and returned from 20 sites. The questionnaires should be filled out, approved prior to inclusion of the first patient and must be updated in case of changes in the treatment procedure.

Selected results from the collected FQs are summarised in Table 3, and some are briefly presented here. Patients are imaged and treated in supine position with arms up at 17 sites, and in supine position with arms down at three sites. The treatment planning systems and dose calculation algorithms used are Eclipse and linear Boltzmann transport equation (seven sites) (Varian Medical Systems, Inc., Palo Alto, CA, USA), RayStation^®^ and Monte Carlo (10 sites), Monaco^®^ and Monte Carlo (one site) (Elekta AB, Stockholm, Sweden) and RayStation^®^ and collapsed cone (two sites) (RaySearch Laboratories AB, Stockholm, Sweden). The prescribed dose is 41.4 Gy in 23 fractions at 10 sites and 50.4 Gy in 28 fractions at 10 sites. Three XT sites use intensity-modulated radiation therapy (IMRT), and seven XT sites use volumetric-modulated arc therapy (VMAT). For the PT sites, four use single-field uniform dose (SFUD), and six sites use intensity-modulated proton therapy (IMPT). Two XT sites use variable beam angles, and five sites use a number of full or partial arcs. One XT site has a class solution consisting of beam angles of 220°, 300°, 0°, 60° and 155° for Truebeam and 220°, 300°, 330°, 0°, 30°, 60° and 155° for Halcyon. Several different robust evaluation scenarios towards setup and range uncertainties are used at the different sites ranging from 6 to 14 scenarios at XT sites and from 12 to 70 scenarios at PT sites. Also, planning target volume (PTV) margins for XT differ from isotropic expansion of the iCTV of five, seven or eight mm to customised margins. All PT sites use the CTV robust concept, except one site that uses the PTV margin. Daily imaging is kV/x-ray based except for one site, where megavoltage (MV) cone-beam computed tomography (CBCT) is used, and setup is performed using either bone match, GTV/CTV/soft tissue match or both bones and soft tissue match for both XT and PT. Three XT sites and three PT sites use fiducial markers. All XT sites and four PT sites use CBCT for daily setup, whilst six PT sites use orthogonal or oblique kilovoltage imaging and markers.

**Table 3:** Selected results on pre-treatment imaging, treatment planning and treatment delivery from the facility questionnaires.

	Photon therapy sites	Proton therapy sites
**Pre-treatment imaging**		
Patient position	Supine with arms up (9)	Supine with arms up (8)
	Supine with arms down (1)	Supine with arms down (2)
Immobilisation device	Wing board, WingStep (3)	Individual vacuum fixation, w/wo thermoplastic mask (5)
	Arm support (1)	Armshuttle Qfix (1)
	Individual vacuum fixation (4)	Wing board, knee support (1)
	None (1)	None (1)
Fiducial markers used	Yes (3)	Yes (3)
	No (7)	No (7)
**Treatment planning**		
Treatment planning system	Eclipse (5)	Eclipse (2)
	RayStation (4)	RayStation (8)
	Monaco (1)	
Dose calculation algorithm	LBTE (5)	LBTE (2)
	Monte Carlo (3)	Monte Carlo (8)
	Collapsed cone (2)	
Dose specification	Dose to medium (8)	Dose to water (8)
	Dose to water (2)	Dose to medium (2)
Prescribed dose	50.4 Gy/28 fractions (5)	
	41.4 Gy/23 fractions (5)	
Treatment technique	IMRT (3)	SFUD (4)
	VMAT (7)	IMPT (6)
Beam arrangement	Variable beam angles (2)	Variable beam angles (7)
	Variable number of full arcs (3)	Class solution (3)
	Variable number of partial arcs (2)	
	Class solution (1)	
	Fixed number of full arcs (1)	
	NA (1)	
Repainting used		Yes (5)
		No (3)
		Patient dependent (2)
PTV/another margin used	Isotropic 5 mm (4)	Isotropic 8 mm (1)
	Isotropic 7 mm (2)	No (9)
	Isotropic 8 mm (2)	
	Customised (2)	
Robust evaluation scenarios	# Scenarios	# Scenarios
	6 (4), 14 (2), NA (4)	12 (1), 14 (1), 16 (1), 20 (1), 28(1), 42(3), 51 (1), 70 (1)
Range uncertainty		3.0% (4)
		3.5% (2)
		4.0% (1)
		5.0% (3)
Setup uncertainty	2 mm isocentre shift (1)	3 mm (1)
	5 mm isocentre shift (5)	5 mm (7)
	7 mm isocentre shift (1)	Other (2)
	NA (3)	
Robustness towards respiration	All phases (4)	All phases (8)
	Inspiration and expiration phase (1)	Inspiration and expiration phase (1)
	NA (5)	NA (1)
**Treatment delivery**		
Model	Truebeam (2)	ProBeam (2)
	Truebeam/Halcyon (3)	Proteus Plus (2)
	Clinac (1)	Proteus ONE (4)
	Versa HD (2)	Proteus 235 (1)
	Tomotherapy (1)	Home built (1)
	Agility (1)	
Imaging for daily setup	kV CBCT (9)	kV CBCT (4)
	MV CBCT (1)	kV/kV and markers (6)
Structure used for setup	Bones (3)	Bones (6)
	GTV/CTV/Soft tissue (6)	CTV (3)
	Bones and soft tissue (1)	Bones and soft tissue (1)

The number in brackets indicates how many sites use the specific technique/device. LBTE: Linear Boltzmann transport equation; IMRT: intensity-modulated radiation therapy; VMAT: volumetric-modulated arc therapy; SFUD: single-field uniform dose; IMPT: intensity-modulated proton therapy; kV: kilovoltage; MV: megavoltage; CBCT: cone-beam computed tomography; GTV: gross tumour volume; CTV: clinical target volume.

On-trial QA: Sixty-four patients have been included in the study from three sites. A total of 22 ICRs have been performed. For the delineation of targets and OARs, six major and 11 minor variations were reported, and for six patients, there were no remarks. For major variations, the extension of CTV *along* the oesophagus and 2 cm into the stomach for distal tumours was the most frequent violation (three patients), followed by not generating the iCTV properly (two patients). For the last case, skin was delineated as 5 mm instead of 10 mm. Major variations were corrected and resubmitted before the third fraction of RT treatment.

The treatment planning ICRs were returned with minor variations in four cases and one major variation. For XT, the mean dose to iCTVTotal (D_mean_) was >101% of the target dose for three patients (secondary constraint: 99% < D_mean_ < 101%). The maximum dose to 0.5 cm^3^ to stomach-iCTV was >103% of the target dose on the XT plan for one patient (secondary constraint). The XT plan of one patient violated the primary constraint of volume of the lungs receiving 5 Gy (V_5Gy_), which should be less than 70% (V_5Gy_ = 96%). This was due to a large target size, iCTVTotal = 612 cm^3^. The treatment planning checklist used for the ICRs is shown in Supplementary material S3.

**Figure 2:**
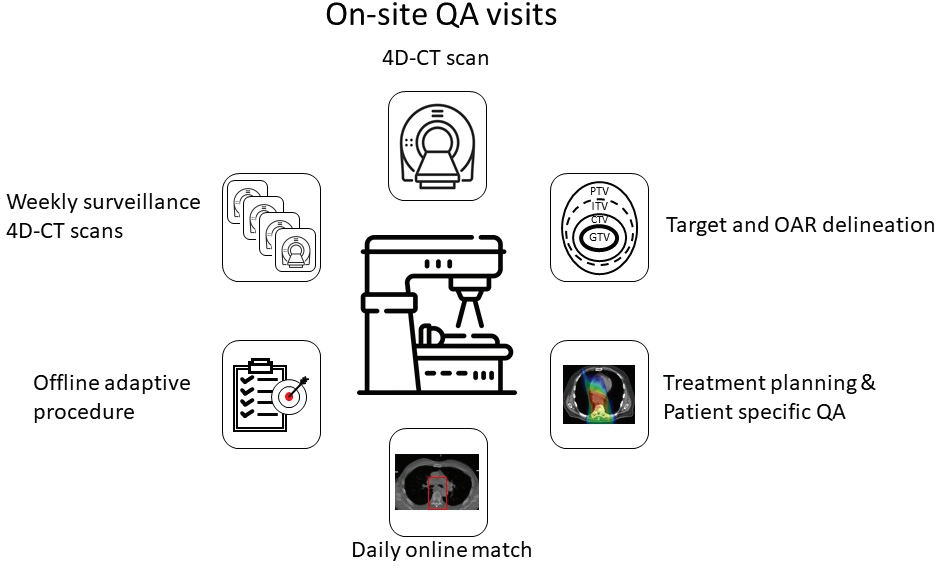
On-site visits are performed when a site/two corresponding sites has included two patients for both PT and XT. A physician and a physicist from the RTQA group will discuss all parts of the treatment process with a radiation oncologist and a physicist and dosimetrist from the site to be audited. XT: Photon therapy; PT: proton therapy; RTQA: radiotherapy quality assurance.

Three site visits have been performed: at Aarhus University Hospital, Department of Oncology and Danish Centre for Particle Therapy (Denmark), at the University Hospitals Leuven and Particle Therapy Interuniversity Centre Leuven (Belgium) and at the University Hospital Zürich and the Paul Scherrer Institute (Switzerland). Simulation CT scanning procedures, delineation, treatment planning, online treatment procedures, offline adaptive therapy, weekly surveillance 4D-CT scanning and patient-specific QA procedures were discussed at all site visits (Figure 2). The members from the RTQA group were present at the simulation CT-scan, dose planning and during treatment delivery of actual patients at each site. Topics like setup margins as well as robust evaluation were discussed at all site visits since both respiratory motion and anatomical changes during the course of RT may have a large impact on the treatment. All centres complied with the guidelines, and only minor comments were submitted to the sites after the visits.

Eighteen sites have submitted beam output audits, which have all been approved. Results from the audit measurements will be presented separately and are briefly summarised here. Measurements were either based on ion chambers, thermoluminescent or optically stimulated luminescent dosimeters. Measurements were all within 3% agreement.

Secondary dose calculation will be installed at sites that presently do not have this opportunity.

Annual workshops: Two annual workshops were held online with 43 and 41 participants. The workshops included 15 and 11 presentations on, e.g., the QA review process, inter-fractional motion, online matching procedures, adaptation and treatment planning from the RTQA group and from external speakers.

## Discussion

In this study, we have described the comprehensive RTQA programme implemented in the PROTECT trial, which encompasses both pre-trial and on-trial RTQA measures. These include benchmark cases, ICRs, FQs, on-site visits, patient-specific QA and beam output audits. The robust RTQA framework implemented in the PROTECT trial has been essential for ensuring consistent treatment delivery across participating centres, as deviations from protocol may compromise the reliability of study findings when patient inclusion ends [29]. Target delineation, in particular, has been shown to be susceptible to deviations, which can significantly affect clinical outcomes. For the target delineations in this study, six major deviations were found of 22 ICRs (27%), consistent with findings from previous studies [16–18, 30]. Importantly, no resubmissions of treatment plans were required, likely due to the stringent pretrial QA process, which included four treatment planning benchmark cases and detailed descriptions of the treatment planning and robustness evaluation provided in the RTQA guidelines. Whilst benchmark cases are important for training of staff at participating sites, deviations can still occur post-training, suggesting that on-trial QA should also be mandatory as recommended by the EORTC [31, 32] and in the paper by Wills et al. on QA of the SCOPE 1 (study of chemoradiotherapy in oesophageal cancer plus or minus erbitux) trial on oesophageal RT [33]. In SCOPE 1, a single benchmark case was utilised to train all participating centres in delineation and treatment planning prior to trial initiation. Despite this standardisation effort, protocol deviations were observed throughout the trial. Given resource limitations, continuous monitoring of delineations and treatment plans was conducted primarily in a retrospective manner. Additionally, the delineation reference standard was determined by two individuals rather than by consensus guidelines. FQs were returned by only 53% of participating centres, and submission was not a prerequisite for the RTQA approval. In the PROTECT trial, we have taken the RTQA process one step further by using several benchmark cases (five for delineation and four for treatment planning), consensus guidelines for delineation and treatment planning, mandatory FQs and beam output audits before patient inclusion, prospective on-trial RTQA of every fifth patient as well as site visits and annual workshops to exchange trial experience.

Deviations from clinical protocols can adversely impact patient outcomes, underscoring the critical importance of RTQA in clinical trials [12]. Despite its significance, there is currently considerable variability in the implementation of RTQA [34]. The RTQA framework used in the PROTECT trial aligns with best practices established in prior studies of radiation therapy in oncology trials [11, 12] and mirrors the approach taken in other multicentre trials, such as the EORTC and CONVERT (concurrent once-daily versus twice-daily chemoradiotherapy in patients with limited-stage small-cell lung cancer) lung cancer trials [9, 14]. Here, pre-trial QA improved compliance with the protocol and thereby improved the reliability of study outcomes. Such QA processes are especially vital in trials involving advanced radiation techniques, where even minor deviations in dose delivery or target delineation may significantly affect patient outcomes [16, 21].

Incorporating FQs into a comprehensive QA program is essential for maintaining protocol compliance and ensuring high-quality treatment delivery across multiple centres [19, 35]. Whilst on-site visits are resource- and time-intensive, they play a pivotal role in allowing RTQA personnel to thoroughly evaluate each stage of the treatment planning process [36]. These visits allow for direct interaction with the clinical staff responsible for patient treatment under the trial protocol, facilitating a comprehensive evaluation of the workflow. Moreover, the establishment of a personal contact during these visits fosters more effective communication and collaboration, which helps address any subsequent issues or deviations from the protocol.

A limitation of the project is that the on-trial ICR is very time-demanding for the clinical personnel involved. This may have caused some centres to decline from participation in the study and may even cause a limited number of patients to be included from the participating sites. Not all patients undergo on-trial RTQA, which is another limitation. In this paper, we have summarised the findings from the inclusion of the first 64 patients (16% of the total patient cohort to be included). The RTQA will continue during the entire trial period, and findings from all sites and all patients will be reported when the trial period ends.

In conclusion, the unique and strict RTQA programme within the PROTECT trial is essential for training and maintaining protocol adherence, minimising deviations and ensuring that the trial’s findings are both reliable and reproducible. Such a rigorous RTQA programme not only improves patient outcomes but also ensures that conclusions drawn from the trial can be applied in broader clinical practice.

## Supplementary Material

Radiotherapy quality assurance in the PROTECT trial – a European randomised phase III-trial comparing proton and photon therapy in the treatment of patients with oesophageal cancer

## Data Availability

The data that support the findings of this study are available from the corresponding author, CSB, upon reasonable request.
