# Supplementary material for: Radiotherapy quality assurance in the PROTECT trial – a European randomised phase III-trial comparing proton and photon therapy in the treatment of patients with oesophageal cancer
Source: Acta Oncol. 2025 Mar 13;64:42774. doi: 10.2340/1651-226X.2025.42774 (PMC11931855; doi:10.2340/1651-226X.2025.42774)
Supplement: Radiotherapy quality assurance in the PROTECT trial – a European randomised phase III-trial comparing proton and photon therapy in the treatment of patients with oesophageal cancer [file AO-64-42774-s1.pdf]

Guidelines for planning and QA of XT and PT in the PROTECT trial

This is a controlled document which should be referred to in conjunction with the PROTECT protocol. All guidelines mentioned here are mandatory for patients enrolled in PROTECT.

XT and PT refer to photon therapy and proton therapy, respectively.

Version 2.0, September, 2023

RTQA Committee:

Christina T. Muijs, Medical doctor, Department of Radiation Oncology, University Medical Center Groningen, University of Groningen, The Netherlands.

Gilles Defraene, Medical physicist, Postdoctoral fellow FWO (Research Foundation Flanders), Laboratory of Experimental Radiotherapy, Department of Oncology, UZ Leuven, Herestraat 49 - box 7003, B - 3000 Leuven, Belgium.

Gloria Vilches Freixas, Medical physicist, PhD; Maastro, Dr Translaan 12, Maastrict, The Netherlands.

Hanna R. Mortensen, Medical doctor, PhD; Aarhus University Hospital, Danish Centre for Particle Therapy, Palle Juul-Jensens Boulevard 99, DK-8200 Aarhus N, Denmark.

Mai L. Ehmsen, Medical physicist, PhD; Aarhus University Hospital, Danish Centre For Particle Therapy, Palle Juul-Jensens Boulevard 99, DK-8200 Aarhus N, Denmark.

Maria F. Jensen, Medical physicist, PhD, Aarhus University Hospital, Danish Centre For Particle Therapy, Palle Juul-Jensens Boulevard 99, DK-8200 Aarhus N, Denmark.

Pieter Populaire, Medical Doctor; KU Leuven – University of Leuven, Department of Oncology – Laboratory of Experimental Radiotherapy; University Hospitals Leuven, Department of Radiation Oncology, B-3000 Leuven, Belgium.

Rebecca Bütof, Medical doctor, PhD; Department of Radiotherapy and Radiation Oncology, Faculty of Medicine, University Hospital Carl Gustav Carus, TU Dresden, Fetscherstr. 74; 01309 Dresden, Germany.

Richard Canters, Medical physicist, PhD; Maastro, Dr Translaan 12, Maastrict, The Netherlands.

RTQA Managers:

Camilla S. Byskov, Medical Physicist, PhD; Aarhus University Hospital, Department of Oncology, Palle Juul-Jensens Boulevard 99, 8200 Aarhus N, Denmark.

Mai L. Ehmsen, Medical Physicist, PhD; Aarhus University Hospital, Danish Centre for Particle Therapy, Palle Juul-Jensens Boulevard 99, 8200 Aarhus N, Denmark.

protect.rtqa@rm.dk

RTQA Chairs:

Lone Hoffmann, Medical Physicist, PhD; Aarhus University Hospital, Department of Oncology, Palle Juul-Jensens Boulevard 99, 8200 Aarhus N, Denmark.

Ditte Sloth Møller, Medical Physicist, PhD; Aarhus University Hospital, Department of Oncology, Palle Juul-Jensens Boulevard 99, 8200 Aarhus N, Denmark.

Table of contents

Table of contents **4**

1. Imaging for delineation and treatment planning **7**

1.1. 4D-CT scanning procedures **7**

1.1.1. Intravenous contrast 7

1.1.2. CT calibration 7

1.1.3. Planning phase or image of 4D-CT used for target delineation 8

1.1.4. Planning phase or image of 4D-CT used for treatment planning 8

1.2. (FDG)-PET scanning procedures **8**

1.2.1. Image registration 8

2. Delineation **8**

2.1. ROIs **8**

2.2. Target volumes **9**

2.2.1. Gross Tumor Volumes 9

2.2.2. Clinical Target Volumes 10

2.2.3. Intra and inter fractional motion 10

2.3. Organs At Risk (OAR) **11**

3. Treatment Planning **13**

3.1. Dose prescription and target coverage **13**

3.2. Treatment plan optimization and dose calculation **13**

3.2.1. XT plans: 13

3.2.2. PT plans: 14

3.3. Target dose constraints **14**

3.4. OAR constraints **15**

3.5. Robustness evaluation before treatment **16**

3.5.1. Robustness towards setup and range uncertainties 16

3.5.2. Robustness towards respiratory motion 17

3.6. Dual dose planning **18**

4. Verification of treatment delivery **18**

4.1. Verification of the daily treatment position **18**

4.1.1. PT centers without CBCT or CT on rails 18

4.2. Adaptive strategy **19**

4.3. Centers with daily CBCT or CT on rails: Tumor and lymph node position **19**

4.4. Centers with daily CBCT or CT on rails: Anatomical changes **19**

4.5. PT centers without CBCT or CT: Position of fiducial markers **20**

4.6. PT centers without CBCT or CT: Anatomical changes **20**

4.7. Adaptation **20**

4.8. Weekly 4D-CT **22**

5. Patient specific QA **23**

6. Protocol variations **23**

7. Quality assurance **24**

7.1. Radiation treatment plan banking **25**

7.1.1. Anonymization 26

7.1.2. Nomenclature 26

7.1.3. Deadlines 27

7.2. Pre-trial QA program **27**

7.2.1. Facility questionnaire 27

7.2.2. Delineation benchmark cases 28

7.2.3. Treatment planning benchmark cases 28

7.2.4. Intra fractional robustness 28

7.2.5. Adaptive strategy 29

7.2.6. Export of benchmark cases 29

7.2.7. Beam Output Audit 29

7.3. On-trial QA program 30

7.3.1. Prospective Individual Case Review (ICR) before treatment start 30

7.3.2. Case review of every fifth patient within one week after treatment start 30

7.4. Prospective trial QA of all patients 30

7.4.1. On-site visit 31

7.4.2. Yearly workshops 31

7.5. Review of weekly 4D-CT scans 31

References 32

1. Imaging for delineation and treatment planning

Delineation and treatment planning require a 4D-CT scan and an FDG-PET scan. The FDG-PET scan may be either 3D or 4D.

1.1. 4D-CT scanning procedures

All 4D-CT scans for planning should fulfill:

- Maximum slice separation 2 mm.
- Minimum 8 phases of the 4D-CT scans have to be reconstructed
- The scan length should include the whole target, the full extension of the lungs, and the full extension of the liver and kidneys if target extends below the diaphragm. If target is at the level of the kidneys, the scan should include the full extension of the kidneys. If one 4D-CT cannot encompass the full length as described above, two 4D-CTs should be acquired and combined into one 4D-CT encompassing the full length
- Patient positioned in the immobilization device used for treatment
- Should be acquired within three weeks prior to the start of XT or PT
- Single or dual energy CT

A 3D-CT alone cannot replace a 4D-CT for treatment planning.

1.1.1. Intravenous contrast

For all patients, a scan with intravenous contrast should be obtained for planning if permitted by patient's renal function. For XT, it can be either a 4D-CT with contrast or a 4D-CT combined with a 3D scan with contrast. For PT, a 4D-CT without contrast is used for planning and supplemented by additional 4D-CT or 3D-CT with contrast. However, if contrast is used at the 4D-CT, HU override should be applied for PT planning.

1.1.2. CT calibration

For all patients treated at a PT center, the CT calibration curve should adhere to the center's best possible standard. Site specific calibration curves based on thorax sized phantom

measurements and beam hardening corrections should be used if it results in more correct dose calculation.

1.1.3. Planning phase or image of 4D-CT used for target delineation

The mid-ventilation phase, the mid-position phase or the average image of the planning 4D-CT scan is used for target and organs at risk delineation.

Treatment in breath hold is not allowed.

1.1.4. Planning phase or image of 4D-CT used for treatment planning

- For XT, the treatment plan should be based on the mid-ventilation phase, the mid-position phase or the average-CT of the planning 4D-CT scan.
- For PT, the treatment plan should be based on the average-CT of the planning 4D-CT scan. The comparative plan, not used for treatment, can be made on the mid-ventilation phase, as long as it is robust for setup and breathing motion.

1.2. (FDG)-PET scanning procedures

- (FDG)-PET scan:
  - Recording time (from start of tracer injection to the (FDG)-PET scan): 60 min +/- 5 min
  - Blood sugar level: <11 mmol/L. Otherwise, the (FDG)-PET scan must be postponed
  - Injection dose: Follow individual center guidelines

1.2.1. Image registration

If the planning 4D-CT and the (FDG)-PET are not acquired simultaneously, the image used for target delineation and the (FDG)-PET should be co-registered.

2. Delineation

2.1. ROIs

For all patients, it is mandatory to delineate the following regions of interest named according to AAPM TGR 263 [1]:

Target structures	Organs at risk (OAR)	
GTVp	Body	Lung_L
GTVn	Bowel_Cavity	Lungs
CTVp	Heart	Skin
CTVn	Kidney_R	Spinal_Cord
CTVtotal	Kidney_L	Spinal_Cord_PRV
iCTV_Dose (Gy)	Kidneys	Spleen
	Liver	Stomach
	Lung_R	

2.2. Target volumes

Delineation of target volumes and organs at risk is based on the consensus guidelines and atlas, in brief summarized below. Further details including atlas are given in ref [2].

2.2.1. Gross Tumor Volumes

GTVp includes the primary tumor (with the esophageal wall) as seen on the planning (FDG)-PET/CT scan and includes all available information (e.g. endoscopy, echo-endoscopy (EUS), diagnostic (FDG)-PET/CT, magnetic resonance imaging (MRI), fiducial markers). The GTVp does not include the peri-esophageal fat, but the entire esophageal wall should be included at the level of the GTVp. If fiducial markers are placed at the tumor borders, they should be included in the GTVp. It is recommended to discuss the GTVp delineation with a radiologist.

GTVn includes lymph nodes defined as pathological any time before XT or PT. Lymph nodes that appear as new on the planning (FDG)-PET/CT compared to the diagnostic (FDG)-PET/CT, suspected to be malignant lymph nodes, have to be included in GTVn. A fine-needle aspiration cytology (FNAC) is recommended in case of doubt, and when it has an impact on the

delineation of the target volume. Delineation of the GTVn is done on the planning (FDG)-PET/CT and includes all available information from e.g. endoscopy, EUS, endobronchial ultrasound (EBUS), diagnostic (FDG)-PET/CT, biopsy, MRI, ultrasound. It is recommended to discuss the GTVn delineation with a radiologist.

2.2.2. Clinical Target Volumes

CTVp includes the GTVp with an expansion of 1.0 cm radially and 3.0 cm cranio-caudally along the esophagus. For tumors in the lower oesophagus and gastro-esophageal junction (GEJ), the CTVp is restricted to 2.0 cm distal to the tumor. The CTVp is corrected for anatomy (muscles, bones, large vessels and OAR) if no invasion.

CTVn includes the GTVn with an expansion of 1.0 cm in all directions and the lymph node stations at this level. Additionally, it includes the lymph nodes stations along the CTVp according to the classification of Hagens et al. [3], including the para-oesophageal lymph nodes (paravertebral 1.0 cm posterior to the anterior border of the vertebral body), the vena azygos, the aortic-pulmonal fenestra, the fatty tissue of the arteria gastrica sinistra, of the subcarinal, para/pretracheal, paracardial and supraclavicular region as far as they are within 3.0 cm cranio-caudally from the GTVp. The CTVn is corrected for anatomy (muscles, bones, large vessels and OAR) if no invasion.

CTVtotal is the sum of CTVp and CTVn. The CTVtotal is expanded to include potential gaps between the CTV's. The potential gaps should always include the esophagus and the lymph nodes station at that level. Depending on the location of the gaps, the para-oesophageal lymph nodes, the aortic-pulmonal fenestra, the fatty tissue of the arteria gastrica sinistra, and the subcarinal, para/pretracheal, paracardial and supraclavicular region should be delineated along the CTVtotal. If the distance of the gap is more than 3.0 cm, the decision to expand the CTVtotal or to irradiate two separate volumes is up to the treating physician.

2.2.3. Intra and inter fractional motion

The intra-fractional motion originates from respiratory and cardiac motion in addition to patient motion. The inter-fractional motion originates from setup errors, target deformation and anatomical changes during the treatment course.

The internal movement originating from the respiratory motion should be accounted for by extending the CTVtotal to iCTVtotal:

- Generate GTVp, GTVn, CTVp, CTVn and CTVtotal as described above.
- **iCTVtotal** is the sum of the CTVtotal in all phases of the 4D-CT scan to account for respiratory motion. The iCTVtotal can include muscles, large vessels and OAR, but bones should be excluded.

The inter-fractional motion can be evaluated on daily imaging scans or weekly CT scans.

The inter-fractional motion originating from setup errors may be accounted for by robust optimization or creation of a PTV margin. Inter-fractional changes beyond those accounted for, should be evaluated and accounted for by use of an adaptive strategy.

2.3. Organs At Risk (OAR)

Delineation of organs at risk

All organs at risk (OAR) are delineated according to the description below. OARs are delineated on mediastinal window (Window Width 400; Window Level 20) unless otherwise specified.

Body is delineated as the patient surface excluding table and any immobilization equipment.

Bowel_Cavity is delineated as the peritoneal space occupied or potentially occupied by bowel excluding vessels, muscles and other organs. The structure should extend at least 1 cm beyond the PTV for treatment plans using coplanar beam only. For non-coplanar beams, the structure should extend 1cm beyond non-coplanar field entrance/exit. The choice depends on the treatment technique [4].

Heart is delineated as the whole heart: Cranially, the superior part of the heart starts just inferiorly to the left pulmonary artery; on an axial CT scan, 10-12mm superiorly to where the pulmonary trunk appears as an independent round structure. Inferiorly, the heart blends with the diaphragm. Since coronary vessels run in the fatty tissue within the pericardium, the pericardium should be included in the heart contours, even if there is no heart muscle visible in that area. The superior vena cava can be included for simplification and consistency [5,6].

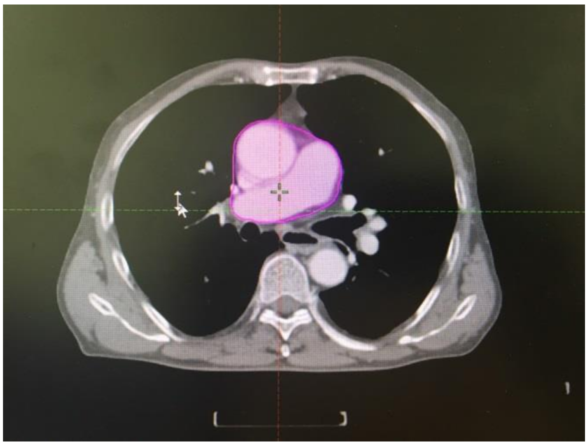

Figure 2.1 Cranial border heart

Kidney_R and Kidney_L are delineated as a whole organ following the boundaries of the kidney excluding the kidney pelvis and ureters [7].

Kidneys are generated by combining Kidney L and Kidney R.

Liver is delineated as the entire liver excluding portal vein and gall bladder. The *inferior vena cava* (IVC) should be excluded from the liver contour when it appears discrete and separate from the liver. The *portal vein* (PV) should be excluded from the liver contour only when segment I is present posteriorly to the PV[8].

Lung_R and **Lung_L** are delineated on lung window (Window Width 1600; Window Level -600). Both lungs are automatically contoured and adapted manually afterwards. All inflated and collapsed, fibrotic and emphysematic parts of the lung should be included as well as small vessels extending beyond the hilar regions; however, hilar and trachea/main bronchus should not be included[9].

Lungs are generated by combining Lung L and Lung R.

Skin is delineated by generating a 10 mm inner margin from the body-outline.

Spinal_cord and PRV spinal_cord: The spinal cord is a circular/oval structure in the canalis spinalis. It is delineated in the whole length, and associated PRV is generated by expanding the spinal cord with 5 mm in all directions.

Spleen is delineated as a whole organ following the boundaries of the spleen[10].

Stomach is delineated as the whole organ from fundus to bulbus ventriculi following the boundaries of the stomach. The substructure **Stomach_fundus** may be contoured separately.

Determine the most proximal part of the stomach within the diaphragmatic dome in the transverse plane. From that point, delineate 12 mm in caudal direction following the boundaries of the stomach [11].

3. Treatment Planning

The planning technique is based on the ICRU62, ICRU78 and ICRU83 recommendations [12-14].

3.1. Dose prescription and target coverage

The treatment plan aims for a homogeneous dose distribution within the target that ensures 41.4 Gy/23 fractions or 50.4 Gy/28 fractions to the iCTVtotal and is prescribed as dose to the iCTVtotal. For PT, Gy should be read as Gy(RBE) with RBE=1.1. Each PT center (and its referring centers) should choose one dose level for all their patients. Sufficient margins, robust optimization, coverage probability or similar strategies should be used to ensure target coverage and will be reviewed during QA. HU-override of iCTVtotal and diaphragm is permitted for dose planning but should be removed for the final dose calculation. If contrast is used, HU override should be applied for PT planning.

3.2. Treatment plan optimization and dose calculation

3.2.1. XT plans:

Inhomogeneity corrections and advanced dose calculation algorithms (Monte Carlo, Acuros, AAA, Collapsed Cone or equivalent) have to be used. Advanced planning techniques such as IMRT, VMAT or equivalent are mandatory. Maximum calculation grid size is $3x3x3mm^3$.

Respiratory motion (excluding interplay effect) is considered through coverage of the iCTVtotal. All other uncertainties (anatomical changes, machine uncertainties, target deformation, delineation etc.) are considered either by:

- Applying a PTV margin. The margin should be based on setup errors determined at each clinic and should reflect the setup strategy selected by the clinic.

- Robust optimization. The setup errors used for the optimization should be based on setup errors determined at each clinic and should reflect the setup strategy selected by the clinic. Other uncertainties may be accounted for by additional input in the robust optimization.
- A combination of robust optimization and iCTVtotal + margin.

3.2.2. PT plans:

Advanced dose calculation algorithms (Monte Carlo, Acuros or equivalent) are mandatory. Patients should be treated with spot scanning technique either as single field uniform dose (SFUD) or intensity modulated proton therapy (IMPT) using 2-3 beams. Beam direction should be posterior/oblique posterior with gantry angles in the range 140 – 220degrees. It is allowed if the same beam angles are obtained through a combination of gantry and couch rotations. The use of lateral beams is not allowed to avoid beam passage through the diaphragm [15,16]. A constant RBE=1.1 is used for treatment planning. Maximum calculation grid size is $3x3x3mm^3$

Robust optimization taking rigid setup errors and range uncertainties into account is mandatory. For the optimization, the planning iso-center should as a minimum be shifted in at least 6 different x, y, and z directions (faces of a cube). Range errors should be included by applying a perturbation on the CT densities, where range may be set to a fixed number (e.g. 3%) or may be the estimated range uncertainty of the beams used for the treatment. The setup errors used for the optimization should be based on setup errors determined at each clinic and should reflect the setup strategy selected by the clinic. A combination of rigid setup errors and iCTV+margin may be used for the optimization [16]. Respiration (excluding interplay effect) is considered through coverage of the iCTVtotal. 4D robust optimization using all respiratory phases for coverage of iCTVtotal is optional. Repainting is optional. Additional uncertainties (anatomical changes, machine uncertainties, target deformation, delineation etc.) can be considered either by a margin or by additional input in the robust optimization.

3.3. Target dose constraints

For the nominal plan, dose constraints for target coverage are given in Table 3.1. First priority constraints are mandatory for all patients. Second priority constraints are not mandatory, but

should be fulfilled if possible. Target coverage (iCTVtotal) is prioritized above OAR dose constraints except for spinal cord constraints.

First priority constraints		
**Target**	**Dose (nominal plan)**	**Dose (all scenarios)**
iCTVtotal	**$V95\%_{iCTVtotal}$ >99%**	**$V95\%_{iCTVtotal}$ >97%**
iCTVtotal	**98% < Dmean < 102%**	
**Second priority constraints**		
**Target**	**Dose (nominal plan)**	**Dose (all scenarios)**
iCTVtotal		**$V95\%_{iCTVtotal}$ >98%**
iCTVtotal	**99% < Dmean < 101%**	

Table 3.1 Dose constraints for nominal plan and all scenarios for robust evaluation of setup and range uncertainties

3.4. OAR constraints

For the nominal plan dose constraints for OARs are given in Table 3.2. First priority constraints are mandatory for all patients. However, if iCTVtotal coverage is insufficient according to table 3.1, the priority of iCTVtotal is prioritized above Lungs and Body constraints. The constraints for spinal cord must always be fulfilled. Second priority constraints are not mandatory, but should be fulfilled if possible.

First priority constraints		
**Organs at risk**	**Dose (nominal plan)**	**Dose (all scenarios*)**
**Spinal_cord**	$D_{0.05cm3}$ < 45Gy	$D_{0.05cm3}$ < 50Gy
**Spinal_cord_PRV**	$D_{0.05cm3}$ < 50Gy	
**Lungs**	MLD < 20Gy<br>V20Gy < 35%	Not evaluated

	V5Gy < 70%	
**Body**	$D_{0.05cm3}$ < 110%<br>D_{1cm3} < 107%	D_{1cm3} < 110%<br>D_{5cm3} < 107%

Second priority constraints

Organs at risk	Dose (nominal plan)	Dose (all scenarios)
**Lungs**	V5Gy < 60%	Not evaluated
**Liver**	V30Gy < 33%	Not evaluated
**Heart**	V40Gy < 30%<br>V25Gy < 50%<br>MHD < 26Gy	Not evaluated
**Kidneys**	MKD < 10 Gy<br>V20Gy < 32%<br>V6Gy < 30% for one functional kidney	Not evaluated
**Bowel_cavity**	V30Gy < 600 cm^3<br>V45Gy < 300 cm^3	Not evaluated
**Stomach - iCTV**	$D_{0.5cm3}$ < 103%	Not evaluated
**Spleen**	MSD < 20Gy	Not evaluated

Table 3.2 Dose constraints for nominal plan and all scenarios for robust evaluation of setup and range uncertainties. For PT, Gy is Gy(RBE) with RBE=1.1. *All scenarios include all scenarios used for setup and range uncertainty and evaluation of respiratory motion.

3.5. Robustness evaluation before treatment

3.5.1. Robustness towards setup and range uncertainties

The robustness towards rigid setup errors and range uncertainties should be calculated on the image used for treatment planning. The robustness should be evaluated by shifting the

planning iso-center in at least 6 different x, y, and z directions (faces of a cube) for both modalities. The shifts should be based on the center-specific inter-fractional errors reflecting setup strategy and should correspond to the uncertainties considered during treatment planning. Range errors should be evaluated for PT plans only by randomly applying a perturbation on the CT densities, where range may be set to a fixed number (e.g. 3%) or may be the estimated range uncertainty of the beams used for the treatment. The combination of setup shifts and range variations constitutes all scenarios to be evaluated for PT.

For both modalities, all scenarios should fulfil constraints given in Table 3.1 for target coverage and Table 3.2 for OARs. This means that the voxel wise worst-case scenario (i.e. each of the scenarios) should fulfil this criterion.

3.5.2. Robustness towards respiratory motion

For both modalities at least one of three scenarios may be used to evaluate the robustness towards respiratory motion. The robustness should be evaluated using the CTVtotal. Each PT and XT center should choose one of the three scenarios:

1. The treatment plan should be recalculated on all respiratory phases of the planning 4D-CT. The re-calculation on each phase should fulfil the constraints given in Table 3.3 for coverage of CTVtotal and in Table 3.2 for OARs.

2. The treatment plan should be recalculated on all respiratory phases of the planning 4D-CT and transferred using a deformable image registration to the image used for treatment planning and accumulated to get the full 4D dose. The 4D dose should fulfil the constraints given in Table 3.3 for coverage of CTVtotal and in Table 3.2 for OARs.

3. The treatment plan should be split on the phases of the 4D-CT preferably using the machine log files (from a dry-run of the treatment plan), recalculated and transferred using a deformable image registration to the image used for treatment planning and accumulated to get the full 4D dose including interplay effect [17]. The 4D dose should fulfil the constraints given in Table 3.3 for coverage of CTVtotal and in Table 3.2 for OARs.

For all patients, the recalculated dose on the expiratory phase and the inspiratory phase must be reported. If scenario two or three is used to evaluate the robustness toward respiration, the accumulated dose must be reported, too.

First priority constraints		
**Target**	**Dose all phases**	**4D dose**
**CTVtotal**	$V95\%_{CTVtotal} > 97\%$	$V95\%_{CTVtotal} > 99\%$

Table 3.3 Dose constraints for CTVtotal for robust evaluation of respiratory motion

3.6. Dual dose planning

Before treatment start, both plans (XT and PT) should be made and should fulfil all planning criteria for both nominal plan and all scenarios for setup and range uncertainties as well as for the plans including the respiratory motion:

- First priority target dose constraints (see Table 3.1 and 3.3)
- First priority OAR constraint (see Table 3.2)

If any of these cannot be fulfilled for both plans, the unavoidable clinical compromises will be accepted, but it will be noted in the clinical report form which of the criteria was violated.

4. Verification of treatment delivery

4.1. Verification of the daily treatment position

Image guidance based on volumetric imaging (enabling soft tissue visualization and registration) should be performed daily before treatment and should be used for matching to ensure high precision of the target position during each treatment fraction. For XT, soft tissue match on the target is preferred for daily setup. However, bone match may be used. For PT, bone match or soft tissue match on the target should be used [18]. Fiducial markers applicable for PT may be used but are not mandatory [19,20].

4.1.1. PT centers without CBCT or CT on rails

In PT centers without the ability to perform daily CBCT or CT, set-up on bones on daily orthogonal kV-images is allowed. In this case, daily evaluation of the position of implanted fiducial markers applicable for PT is mandatory. Surgical clips cannot be used. If the position

of the markers deviates above the preset clinical tolerance, a CT scan should be acquired and used to evaluate the need for adaptation.

4.2. Adaptive strategy

The images acquired for daily image guidance must be used to verify the inter fractional reproducibility of the target positioning. Each department must have a treatment adaptation strategy. The strategy and tolerance limits for target and OARs should be based on margins or uncertainties as determined by the individual center. In case the tolerance limits are exceeded, action based on the adaptive strategy of the treating XT or PT center must be performed.

As an example, for an XT treatment where setup errors of e.g. 5 mm are used in the calculation of the PTV margin, or a PT treatment where the plan is made robust towards setup errors of 5 mm, systematic errors above 5 mm should lead to treatment adaptation. Examples of clinically implemented strategies can be provided by the QA group before center initiation.

4.3. Centers with daily CBCT or CT on rails: Tumor and lymph node position

The images acquired for daily image guidance must be used to verify that the positions of the primary tumor and the treated lymph nodes in three dimensions are within the tolerance limits set by the individual XT or PT center. The tolerances can be different for the tumor target and the lymph node targets. The verification should be performed daily online.

If the position of the primary tumor or the position of the lymph nodes differs systematically from that at planning and beyond the tolerances of the treating center, the treatment plan must be adapted. Adaptive treatment plans are not required in case of tumor shrinkage, unless the dose to the OARs exceeds the tolerance set in Table 3.2. If the patient treatment plan is adapted, shrinkage will be considered only where anatomical borders ensure safe shrinkage of the delineated target.

4.4. Centers with daily CBCT or CT on rails: Anatomical changes

The daily images should also be used to detect major anatomical changes in the thoracic or abdominal region and large changes in the density of the lungs due to e.g. atelectasis, pleural

effusion, or pneumonia / pneumonitis. If the changes are systematic and lead to a markedly modified dose distribution that either exceeds the normal tissue constraints or leads to a risk of iCTVtotal under dosage, the treatment plan must be adjusted. The anatomical changes should be evaluated daily online, whereas the dosimetric consequences can be evaluated off-line.

4.5. PT centers without CBCT or CT: Position of fiducial markers

The images acquired for daily image guidance must be used to verify that the positions of the fiducial markers (representing the primary tumor and the treated lymph nodes) are within the tolerance limits set by the individual PT centers. If the position of the markers deviates above the preset clinical tolerance, a CT scan should be acquired and used to evaluate the need for adaptation.

4.6. PT centers without CBCT or CT: Anatomical changes

For centers without CBCT or CT on rails, the anatomical changes will be picked up partially by the fiducial markers as described above and by the weekly 4D-CT scans mandatory for all patients as described in section 4.8. For the patients treated at PT centers without CBCT, major anatomical changes in the thoracic or abdominal region or large changes in the density of the lungs due to e.g. atelectasis, pleural effusion, or pneumonia / pneumonitis (not distinguishable on planar imaging) will be detected by the weekly 4D-CTs. As described in section 6.5, if a center can show that anatomical changes are caught by the variations in the position of the implanted fiducial markers, weekly 4D-CT can be omitted.

4.7. Adaptation

In case an adaptive treatment plan is indicated, either a new 4D-CT will be acquired or the latest weekly 4D-CT scan may be used if representative. The targets, lungs, heart and spinal cord should be propagated to the new/latest weekly 4D-CT and corrected by a radiation oncologist. Other OARs should always be propagated but only corrected if deemed necessary by the treating physician.

The treatment plan is then recalculated using the new/latest weekly 4DCT scan and iCTVtotal and OARs for the **nominal plan** are evaluated according to Table 4.1.

The effect of residual setup error and range uncertainty on the iCTVtotal should be evaluated according to Table 4.1 (**all scenarios**) using 2 mm set up error and the same range uncertainty as used for evaluation of range uncertainty on the original treatment plans. The evaluation should follow the procedures described in section 3.5.1.

Furthermore, the effects of the respiratory motion on CTVtotal should be evaluated according to Table 4.2 following the procedure described in Section 3.5.2. Either the dose on all phases should be evaluated or the 4D dose. If the tolerances defined in Table 4.1 and Table 4.2 are exceeded, an adaptive plan should be made. However, if only the maximum dose (D_{1cc}) to the body is above threshold, the treating physician may decide that adaptation is not required based on a clinical decision. For all other deviations, adaptation must be performed.

If adaptation is required based on the nominal plan, effect of residual setup error, range uncertainty and respiratory motion does not need to be evaluated, as a new adapted treatment plan must be made.

	**Fraction 1-10**	**Fraction 11-21**	**Fraction 21-end#**
**iCTVtotal Nominal plan**	$V95\%_{iCTVtotal} > 98\%$	$V95\%_{iCTVtotal} > 97\%$	$V95\%_{iCTVtotal} > 96\%$
**All scenarios**	$V95\%_{iCTVtotal} > 96\%$	$V95\%_{iCTVtotal} > 95\%$	$V95\%_{iCTVtotal} > 94\%$
**Spinal cord Nominal plan**	$D_{0.05cm3} < 50Gy$	$D_{0.5cm3} < 50Gy$	$D_{1cm3} < 50Gy$
**Body Nominal plan**	$D_{1cm3} < 110\%$	$D_{2cm3} < 110\%$	$D_{3cm3} < 110\%$
	$D_{5cm3} < 107\%$	$D_{7cm3} < 107\%$	$D_{9cm3} < 107\%$

Table 4.1 Dose constraints for OARs refers to a nominal plan with a full dose prescription. OARs are only evaluated for the nominal plan. # Only relevant for patients treated with 28 fractions.

	Fraction 1-10	Fraction 11-21	Fraction 21-end#
**CTVtotal All phases**	V95%$_{CTVtotal}$ >96%	V95%$_{CTVtotal}$ >95%	V95%$_{CTVtotal}$ >94%
**4D dose**	V95%$_{iCTVtotal}$ >98%	V95%$_{iCTVtotal}$ >97%	V95%$_{iCTVtotal}$ >96%

Table 4.2 Dose constraints for CTVtotal for robust evaluation of respiratory motion

If an adaptive plan is required, the plan should be made according to the instruction for treatment planning in Section 3 fulfilling dose constraints specified in Table 3.1, Table 3.2 and Table 3.3. Robustness toward setup and range uncertainties and respiratory motion should be evaluated. Adaptation should be performed within 2 working days.

4.8. Weekly 4D-CT

4D-CT scans should be acquired weekly (approximately at fraction 5, 10, 15 and 20). The 4D-CT at fraction 20 is optional. The targets, lungs, heart and spinal cord should be propagated to the weekly 4D-CT and corrected by a radiation oncologist. Other OARs should always be propagated but only corrected if deemed necessary by the treating physician.

The treatment plan is then recalculated using the this weekly 4DCT scan and iCTVtotal and organs at risk for the **nominal plan** are evaluated according to Table 4.1.

The effect of residual setup error and range uncertainty on the iCTVtotal should be evaluated according to Table 4.1 (**all scenarios**) using 2 mm set up error and the same range uncertainty as used for evaluation of range uncertainty on the original treatment plans. The evaluation should follow the procedures described in section 3.5.1.

Furthermore, the effects of the respiratory motion on CTVtotal should be evaluated according to Table 4.2 following the procedure described in Section 3.5.2. Either the dose on all phases should be evaluated or the 4D dose.

If any of the criteria in Table 4.1 and Table 4.2 (target coverage, dose limits to OARs, and robustness towards respiration) fail to be fulfilled, a new treatment plan preferably with unchanged beam angles and technique should be made fulfilling all criteria. However, if only the maximum dose (D_{1cc}) to the body is above threshold, the treating physician may decide that adaptation is not required based on a clinical decision. For all other deviations, adaptation must be performed.

This calculation on the weekly 4D-CT scan should be performed within 1 working day. Adaptation should be performed within 2 working days. This gives a maximum of 3 days from acquisition of the 4D-CT to initiation of a potential adaptive plan.

If anatomical changes are observed on a weekly 4D-CT, former 4D-CT scans and CBCT scans should be consulted in order to determine if the deviations are systematic. Only changes deemed to be systematic should be corrected for by an adaptive treatment plan. Furthermore, the dose already delivered during the treatment course should be considered when decision for adaptation is taken. Less frequent 4D-CT scanning can be allowed if approved by the QA group as described in section 7.5.

5. Patient specific QA

Patient specific QA is mandatory, and all treatment plans should pass QA procedures with a γ(5%, 3mm) above 95% or γ(3%, 3mm) above 90%. The γ evaluation must be based on measurements of the treatment plans at an accelerator. In centers routinely using log-files to analyse the treatment plans, this evaluation can substitute the patient specific QA.

Additionally, all treatment plans must pass evaluation in a secondary treatment planning system with γ(5%, 3mm) above 95% or γ(3%, 3mm) above 90%.

6. Protocol variations

A major protocol variation regarding target definition is defined as any case with the risk of violating target coverage throughout the treatment course. This includes but is not restricted to:

- Failure to generate an iCTVtotal
- Not including the entire esophagus, where the CTV includes the GTV
- GTV not being within 10 mm of the propagation described on diagnostic CT, PET-CT or EUS will be discussed with the treating physician. If no substantial clinical reason, the GTV should be corrected. Smaller GTV will be considered a major variation and larger GTV a minor variation

- Less than 10 mm margin to metastatic lymph nodes unless shaping towards bone, vessels etc.
- Not adding the described margins
- Scan range not sufficient according to section 1.1
- Not delineating all OARs included in the scan according to section 1.1 or not delineating OARs in the whole organ extent

Minor variations are defined as all other deviations from consensus guidelines due to clinical decision.

If any major delineation variations are found during QA, the center will be asked to correct the delineation. If minor variations are found, no action will be taken.

Violation of a first priority constraint (Table 3.1 and Table 3.2) for the nominal plan is considered a major protocol variation except if this is deemed clinically necessary. Violation of a second priority constraint (Table 3.1 and Table 3.2) for the nominal plan is considered a minor protocol variation. Furthermore, variations of a first or second priority constraint (Table 3.1, Table 3.2 and Table 3.3) for the evaluation of robustness toward respiratory motion or setup and range uncertainties are considered minor variations. The center will be asked to re-optimize the plan within 3 fractions if major variations are seen for the treatment plan or for the robust evaluation. Only if the variation persists will it be counted as a major variation. No actions will be taken for minor variations.

Failure to adapt patients based on the repeated 4D-CTs, where the tolerances defined in Table 4.1 and Table 4.2 are exceeded, is considered a major variation.

Failure to obtain 4D-CT scans for planning is considered a major variation.

Failure to obtain weekly CT scans is considered a major variation, if the center has no agreement with the QA committee upon this subject

7. Quality assurance

State of the art quality assurance is essential for running modern multi-center XT and PT clinical trials to avoid any delineation or planning uncertainties or errors which may corrupt the primary endpoint. It is considered a major variation if the total treatment time exceeds the allowed limit. The QA group will continuously monitor major and minor variations.

The QA group is responsible for the full QA program described below.

7.1. Radiation treatment plan banking

An overview of data to be exported to the treatment plan bank, ePeerReview, is given in Table 7.1

	CT images	Nominal plan:<br>Plan +<br>Dose cube	Uncertainty evaluation:<br>DVH	PET
Treatment planning	4D-CT<br>AVG[#]	PT and XT	Set up+range[&]<br>(iCTVtotal, Spinal_cord, Body)<br><br>Respiration inspiration+expiration[%]<br>(CTVtotal, Spinal_cord, Body)	Diagnostic[¤]<br>Planning
Weekly 4D-CT and adaptation	4D-CT<br>AVG[#]	PT or XT *	Set up+range[&]<br>(iCTVtotal, Spinal_cord, Body)<br><br>Respiration inspiration+expiration[%]<br>(CTVtotal, Spinal_cord, Body)	

Table 7.1. Data to be exported to ePeerReview. *Only the plan from the modality actually treating the patient. [¤]if acquired. [#] if used for planning. [&]range only for PT. [%]If 4D dose is used for robustness evaluation, DVH for this dose calculation must be uploaded too.

Both treatment plans (XT and PT) for all patients included in the study must be exported to the study imaging and treatment plan bank ePeerReview (secure server address) and used for the QA program described below and for the final analysis. The data should be in DICOM format and should include full 4D-CT scan, average-CT scan, FDG-PET CT scan, structure set, XT and PT plan, XT and PT dose cube for the nominal plan.

The weekly 4D-CT scans should be exported including all phases, adjusted structure sets, and re-calculated XT or PT plans and dose cubes for the nominal plans. Only plans for the modality actually treating the patient should be made and sent.

If one or more adaptive treatment plans are used for treatment, the recalculated plan and dose cube based on the adaptive plan for the modality used for treatment should also be exported. Only plans for the modality actually treating the patient should be made and sent.

DVH parameters in text-format for iCTVtotal and OARs for the nominal plan and all robust evaluation scenarios for the treatment plan, weekly 4D-CT recalculations and adaptive plans used for treatment should be exported.

The worst-case scenario treatment plans (i.e. recalculation of the plan at the inspiration and expiration phases), should also be exported either as dose cube or as DVH parameters in text format. If dose accumulation has been performed (section 3.5.2), the accumulated dose calculated using the full 4D dose should also be exported either a dose cube or as DVH parameters in text format.

The patients must give signed informed consent to participate in the study, including acceptance that dose plan and scans after anonymization will be stored in the dose plan bank ePeerReview. All data in the imaging and treatment plan bank will be available for the QA group and for individuals assigned by the steering committee study board to analyze data after signed agreement between the centers.

7.1.1. Anonymization

All digital data uploaded should be anonymized with respect to name, date of birth and patient ID. The patient ID should be replaced with the patient study ID.

7.1.2. Nomenclature

The nomenclature for target and organs at risk are described in section 2.1. For treatment plans, clearly distinct names should be chosen for the XT and the PT plan. The naming must be consistent within a PT center and all its referring XT centers. In the case of re-planning, the fraction from which the plan is used clinically should be a part of the plan name. For scans, the acquisition date should be part of the series description.

Nomenclature		
	Planning	Adaptation
CT scan	CT0_ddmmyy	CT1_ddmmyy
Structure set	CT_0Gy	CT_xGy (x=Delivered dose@CT scan time)
Plan	Esophagus	Fz_Esophagus (z=fraction number for start of new plan)

Table 7.2 Examples of nomenclature. Other nomenclature can be used, but the naming must be consistent within a PT center and all its referring XT centers

7.1.3. Deadlines

For the first two patients included at each center, all data relevant for case review should be uploaded maximum two working days before treatment start. Data must be reviewed by the QA group within one working day.

For the remaining patients both treatment plans should be uploaded before treatment start.

For the Individual Case Review (ICR) of every fifth patient, data will be reviewed within one week.

All remaining data related to XT or PT should be uploaded within two months after treatment start.

7.2. Pre-trial QA program

The pre-trial QA program consists of several steps described below. All steps should be approved by the QA group before a center can start randomization.

7.2.1. Facility questionnaire

The facility questionnaire must be filled out. The questionnaire will be reviewed annually and must also be updated within two months in case of major changes.

7.2.2. Delineation benchmark cases

All participating centers (PT and each referring XT center) have to participate in a delineation workshop. Five anonymized patient cases used for the consensus guidelines have to be delineated and will be discussed at the workshop. The data will consist of full 4D-CT, FDG-PET CT and clinical description. Centers will be asked to delineate target and OARs according to the delineation guidelines described in section 2.2 and 2.3. The delineations will be compared to the consensus delineations achieved between six esophageal cancer experts from five different centers. If variations, deemed unacceptable by the QA group, are observed, feedback will be provided, and the center in question will be asked to adjust the delineation or delineate on an alternative patient.

7.2.3. Treatment planning benchmark cases

Anonymized data for four protocol-compliant treatment planning benchmark cases will be provided. The data will consist of a 4D-CT scan including an average image with full target and OARs delineations. Centers will be asked to make XT and PT plans according to the treatment planning guidelines described in section 3. All referring XT centers must make 4 XT plans. Both plans should be recalculated in all scenarios accounting for set-up errors and range uncertainties, fulfilling the criteria described in Table 3.1, Table 3.2 and Table 3.3. If large variations, deemed unacceptable by the QA group, are seen, feedback will be given to the center, and the center will be asked to adjust the treatment plans.

7.2.4. Intra fractional robustness

Each center (PT and each referring XT center) should present written guidelines for their workflow and tools to calculate the treatment plan on all respiratory phases of the planning 4D-CT. If the center has chosen to calculate the full 4D dose eventually including interplay effect, guidelines for this procedure should also be presented.

For each of the four patient cases provided above, the calculations should be performed and presented.

7.2.5. Adaptive strategy

Each center should present written guidelines for their strategy for ensuring target coverage (margins, robust optimization or a combination) as well as their adaptive strategy.

For each of the four patient cases provided above, a 4D-CT acquired at fraction 10 will also be provided including re-delineation of all structures. Both XT and PT plan should be re-calculated on this scan. Both plans should also be evaluated for robustness using this scan according to the procedure described in section 4.7 for adaptive procedures.

The results will show the robustness towards real anatomical changes and should be interpreted according to Table 4.1 and 4.2.

7.2.6. Export of benchmark cases

Data from the four benchmark cases should be exported to the imaging and treatment plan bank ePeerReview. The data should be in DICOM format and should include XT and PT plan, XT and PT dose cube for the nominal plan.

For the CT scan acquired at fraction 10, the XT and PT plan, XT and PT dose cube for the nominal plan should be exported.

DVH parameters should be exported in text-format for iCTVtotal and OARs for the nominal plan and for all robust evaluation scenarios for the treatment plan, and the plan recalculated on the CT scan acquired at fraction 10.

For intrafractional motion evaluation, the worst-case scenario (i.e. recalculation of the plan at the inspiration and expiration phases) should be exported. If the center has chosen to calculate the full 4D dose eventually including interplay effect, this plan should also be exported. DVH parameters for CTVtotal and OARs in text format should be exported.

7.2.7. Beam Output Audit

For XT a Beam Output Audit should be performed and authorized. Only Beam Output Audits accepted by EORTC will be accepted. For further information, see https://www.eortc.org/quality-assurance/rtqa/beam-output-audit-boa/. For PT, local

dosimetry audits approved by the EORTC will be accepted. The Beam Output Audit will have to be performed once during the timeframe of the project.

7.3. On-trial QA program

7.3.1. Prospective Individual Case Review (ICR) before treatment start

For the first two consecutive patients treated by a PT center and the first two consecutive patients treated by each XT referring center, prospective ICRs will be performed in two steps:

- 4D-CT, PET-CT, structure set and clinical description should be up-loaded and reviewed by the QA group. The QA group may ask for correction of target or OARs delineations before approval by the QA group. If feasible, treatment planning should await approval of the delineations.
- Dual treatment plans (XT and PT) should be uploaded including calculation of all scenarios regarding set-up errors and range uncertainties as well as the respiratory robustness analysis (see section 3.5). The ICR will evaluate both plans.

7.3.2. Case review of every fifth patient within one week after treatment start

Full case review of every fifth patient included at each PT and each XT center will be performed as described above (section 6.3.1). Full data including structure set, 4D-CT, PET-CT, dual treatment planning and robustness evaluation should be up-loaded.

7.4. Prospective trial QA of all patients

The QA group will perform continuous QA of the treatment plans. This will consist of an automatic monitoring of all uploaded treatment plans. If any major variations of the constraints are found, the QA group will be notified and a complete case review performed. In case of major protocol variations or repeated minor variations (section 6), the QA group will contact the center, and a prospective ICR could be introduced for the next patient treated by the center.

The continuous QA program will also be used to monitor the difference in dose to OARs in the two arms. If variations in the dose difference are expected to affect the primary endpoint power calculation, the QA group will notify the primary investigator.

7.4.1. On-site visit

When each PT and each XT center has enrolled and started treatment of the first two patients in the protocol, a medical physicist and an oncologist from the QA group will perform an on-site visit to monitor:

- Compliance with delineation guidelines (section 2)
- Compliance with planning and evaluation guidelines (section 3)
- Compliance with setup strategy guidelines (section 4.1)
- Compliance with adaptive strategy guidelines (section 4.2)
- Full clinical workflow

After the visit, the QA group will make a written evaluation of the center. If the guidelines are found not to be followed, the center has to adjust their strategy to ensure compliance.

7.4.2. Yearly workshops

A workshop will be held yearly at alternating PT centers. Participants from all activated centers will be invited.

Agenda for the meetings will be:

- Presentation by the QA committee of the continuous QA work
- Presentation of treatment plans, delineations and re-calculation on weekly 4D-CT scans for approximately every tenth patient treated in a one-year period since last workshop. Patient-cases will be selected by the QA group and presented by each PT center (or a referring XT center) amongst patients treated at the PT center and their referring XT centers
- Presentation of challenging patient cases. All centers can present these cases
- Amendments to the protocol

7.5. Review of weekly 4D-CT scans

When a PT or an XT center has treated at least 8 patients and acquired weekly 4D-CT scans, a validation study may be conducted. The study should investigate if less frequent imaging or CBCT-based triggering of adaptations can replace the weekly 4D-CT while maintaining the

same level of sensitivity to anatomical changes. Based on the validation, the QA group can decide to allow a less frequent imaging for that PT or XT center.

If a center has performed the analysis before entering the trial, the QA group can decide to allow a less frequent imaging.

References

[1]https://www.aapm.org/pubs/reports/RPT_263.pdf, https://www.aapm.org/pubs/reports/RPT_263_Supplemental/

[2] Hagens ERC, Van Berge Henegouwen MI, Van Sandick JW, Cuesta MA, Van Der Peet DL, Heisterkamp J, et al. Distribution of lymph node metastases in esophageal carcinoma [TIGER study]: Study protocol of a multinational observational study. BMC Cancer. 2019;19(1):1–8.

[3] Thomas M, Mortensen HR, Hoffmann L, et al. Proposal for the delineation of neoadjuvant target volumes in oesophageal cancer. Radiother Oncol. 2021;156:102-112.

[4] Gay HA, Barthold HJ, Michalski JM. Et al. Pelvic normal tissue contouring guidelines for radiation therapy: a Radiation Therapy Oncology Group consensus panel atlas. Int J Radiat Oncol Biol Phys. 2012;83:e353-62.

[5] Feng M, Moran JM, Pierce LJ et al. Development and validation of a heart atlas to study cardiac exposure to radiation following treatment for breast cancer. Int J Radiat Oncol Biol Phys. 2011;79:10-18.

[6] Duane F, Aznar MC, Taylor CW et al. A cardiac contouring atlas for radiotherapy. Radiother Oncol 2017;122:416-422.

[7] Dawson LA, Kavanagh BD, Schultheiss TE et al. Radiation-associated kidney injury. Int J Radiat Oncol Biol Phys. 2010;76:108-115.

[8] Jabbour Sk, Hashem SA, Dawson LA et al. Upper abdominal normal organ contouring guidelines and atlas: a Radiation Therapy Oncology Group consenses. Pract Radiat Oncol. 2014;4:82-89.

[9] Kong F-M, Quint L, Machtay M, Bradley J. Atlases for Organs at Risk (OARs) in Thoracic Radiation Therapy [Internet]. 2013. Available from: http://www.rtog.org/CoreLab/ContouringAtlases/LungAtlas.aspx%5Cnpapers3://publication/uuid/3E06737D-3F53-49F8-9133-339E3AABA706

[10] Trip AK, Sikorska K, Verheij M et al. Radiation-induced dose-dependent changes of the spleen following postoperative chemoradiotherapy for gastric cancer. Radionther Oncol 2015;116:239-24.

[11] Goense L, van Rossum PSN, van Hillegersberg R et al. Radiation to the gastric fundus increases the risk of anastomotic leakage after esophagectomy. Ann Thorac Surg 2016;102:1798–804.

[12] ICRU Report 62. Prescribing recording and reporting photon beam therapy (supplement to ICRU report 50). International Commission on Radiation Units and Mesurements.

[13] ICRU Report 78. Prescribing, Recording, and Reporting Proton-Beam Therapy. International Commission on Radiation Units and Measurements.

[14] Report 83: Prescribing, Recording, and Reporting Photon-Beam Intensity-Modulated Radiation Therapy (IMRT). International Commission on Radiation Units and Measurements.

[15] Yu J, Zhang X, Liao L, Li H, Zhu R, Park PC, et al. Motion-robust intensitymodulated proton therapy for distal esophageal cancer. Med Phys 2016;43:1111–8.

[16] Validation of a Robust Strategy for Proton Spot Scanning for Oesophageal Cancer in the Presence of Anatomical Changes. Møller DS, Alber M, Nordsmark M, Nyeng TB, Lutz CM, Hoffmann L Radiother Oncol. 2019;131:174-178.

[17] Ribeiro CO, Meijers A, Korevaar EW, Muijs CT, Both S, Langendijk JA, Knopf A. Comprehensive 4D robustness evaluation for pencil beam scanned proton plans. Radiother Oncol. 2019;136:185-189.

[18] Bone versus soft-tissue setup in proton therapy for patients with oesophageal cancer. Shamshad M, Møller DS, Mortensen HR, Ehmsen ML, Jensen MF, Hoffmann L. Acta Oncol. 2022;61:994-1003. doi: 10.1080/0284186X.2022.2091949. PMID: 35775236.

[19] Utility of fiducial markers for target positioning in proton radiotherapy of oesophageal carcinoma. Apolle R, Brückner S, Frosch S, Rehm M, Thiele J, Valentini C, Lohaus F, Babatz J, Aust DE, Hampe J, Troost EGC. Radiother Oncol.2019;133:28-34.

[20] Setup strategies and uncertainties in esophageal radiotherapy based on detailed intra- and interfractional tumor motion mapping. Hoffmann L, Poulsen PR, Ravkilde T, Bertholet J, Kruhlikava I, Helbo BL, Schmidt ML, Nordsmark M. Radiother Oncol. 2019;136:161-168.

Item	Checked	No remarks	Minor remarks	Major remarks	Comment
**Organs at Risk**					
Body	☐	☐	☐	☐	
Bowel__Cavity	☐	☐	☐	☐	
Heart	☐	☐	☐	☐	
Kidney_R	☐	☐	☐	☐	
Kidney_L	☐	☐	☐	☐	
Kidneys	☐	☐	☐	☐	
Liver	☐	☐	☐	☐	
Lung_R	☐	☐	☐	☐	
Lung_L	☐	☐	☐	☐	
Lungs	☐	☐	☐	☐	
Skin	☐	☐	☐	☐	
Spinal_Cord	☐	☐	☐	☐	
Spinal_Cord_PRV	☐	☐	☐	☐	
Spleen	☐	☐	☐	☐	
Stomach	☐	☐	☐	☐	
**Targets**					
GTVp	☐	☐	☐	☐	
GTVn	☐	☐	☐	☐	
CTVp	☐	☐	☐	☐	
CTVn	☐	☐	☐	☐	
CTVtotal	☐	☐	☐	☐	
iCTVtotal	☐	☐	☐	☐	
**Final review**					
Comments					

Plan QA Physics Checklist

The following should be filled out by the dose planner:

	Task	XT	PT
1	Average or mv-scan used for planning		
2	Dose calculation algorithm		
3	PTV and PRV margins		
4	Setup and range (PT) uncertainties used		
5	Overall Dmax (Gy) of the plan		

Approved: ☐ yes ☐ no
Explanation:
Reviewed by:
Date:

The following should be filled out by the RTQA physicist:

	Task	Verified	Major deviations	Minor deviations
1	Patient position	☐ XT<br>☐ PT		
2	Dose grid	☐ XT<br>☐ PT		
3	Special densities and overrides	☐ XT<br>☐ PT		
4	Dose prescription	☐ XT<br>☐ PT		
5	Beam arrangement and isocenter position	☐ XT<br>☐ PT		
6	Dose distribution	☐ XT<br>☐ PT		

Evaluations of dose constraints for XT plan:

First priority constraints of the nominal XT plan:

Structure	Indicator	Value	Constraint
iCTVtotal	Dmean	✓ 100.9127	98% < x < 102%
iCTVtotal	V95%	✓ 100.0000	> 99%
Spinal_Cord	D0.05cc	✓ 41.6758	< 45Gy
Spinal_Cord_PRV	D0.05cc	✓ 44.1957	< 50Gy
Lungs	MLD	✓ 5.9600	< 20Gy
Lungs	V20Gy	✓ 10.8300	< 35%
Lungs	V5Gy	✓ 25.5700	< 70%
BODY	D0.05cc	✓ 104.3650	< 110%
BODY	D1cc	✓ 104.3643	< 107%

Second priority constraints of the nominal XT plan:

Structure	Indicator	Value	Constraint
iCTVtotal	Dmean	✓ 100.9127	99% < x < 101%
Lungs	V5Gy	✓ 25.5700	< 60%
Liver	V30Gy	✓ 13.3300	< 33%
Heart	MHD	✓ 14.4200	< 26Gy
Heart	V40Gy	✓ 13.5300	< 30%
Heart	V25Gy	✓ 23.9400	< 50%
Kidneys	MKD	✓ 2.1900	< 10Gy
Kidneys	V20Gy	✓ 1.6000	< 32%
Kidney_R	V6Gy	✓ 0	< 30%
Kidney_L	V6Gy	✓ 16.4200	< 30%
Bowel_Cavity	V30Gy	✓ 5.0200	< 600cc
Bowel_Cavity	V45Gy	✓ 1.1100	< 300cc
Stomach - iCTV	D0.5cc	! 103.5134	< 103%
Spleen	MSD	✓ 15.1100	< 20Gy

Constraints of all XT scenarios for robust evaluation:

Structure	Indicator	Constraint	Comment
Spinal_Cord	D0.05cc	< 50Gy	✓
BODY	D1cc	< 110%	✓
BODY	D5cc	< 107%	! 108.92Gy for i_00, 107.54Gy for i_10, 108.73Gy for i_90
CTVtotal	V95%	> 97%	✓

Constraints of all XT scenarios for range uncertainties:

Structure	Indicator	Constraint	Comment
Spinal_Cord	D0.05cc	< 50Gy	✓
BODY	D1cc	< 110%	✓
BODY	D5cc	< 107%	! 210.71% for Es_U1, 209.13% for Es_U10, 211.31% for Es_U11, 209.32% for Es_U12,...
CTVtotal	V95%	> 97%	✓

Plot of constraints of all XT scenarios for robust evaluation:

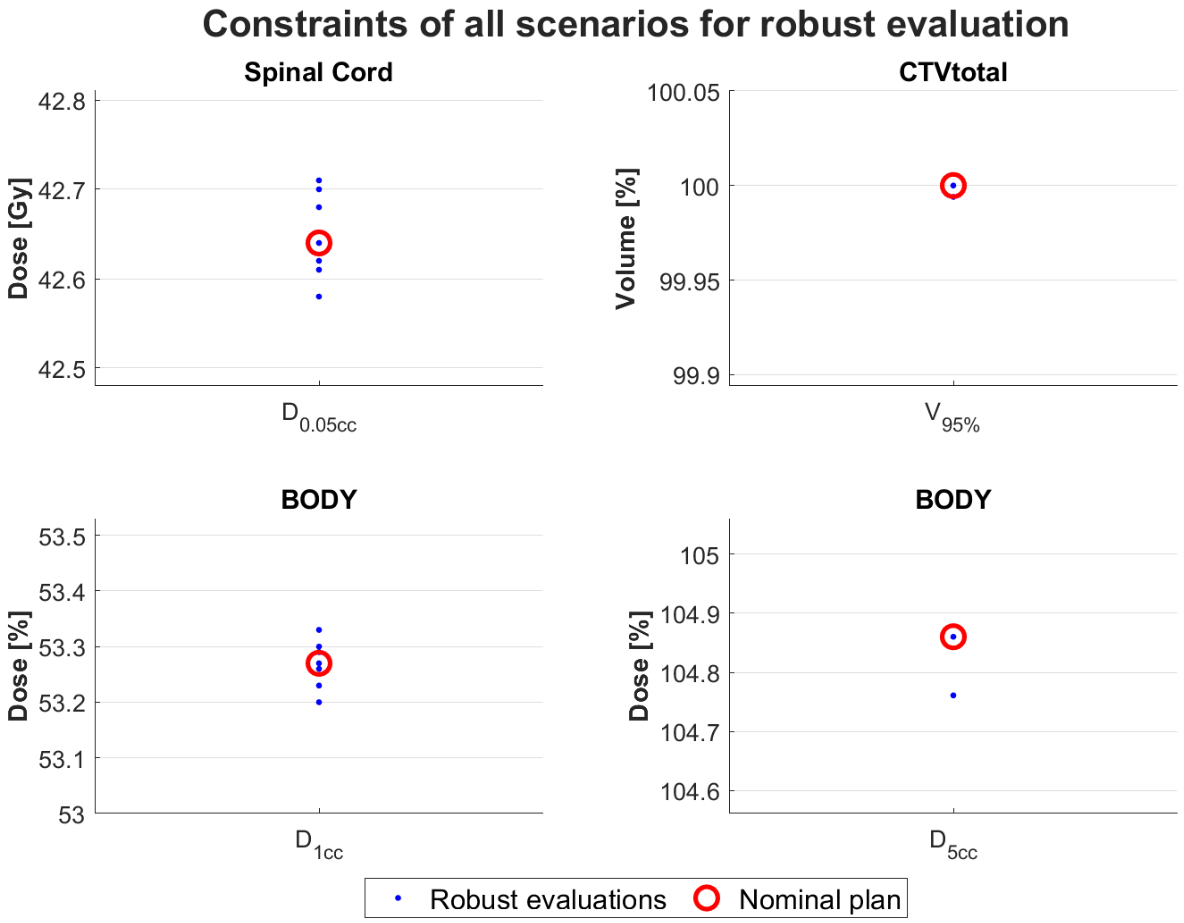

Plot of constraints of all XT scenarios for range uncertainties:

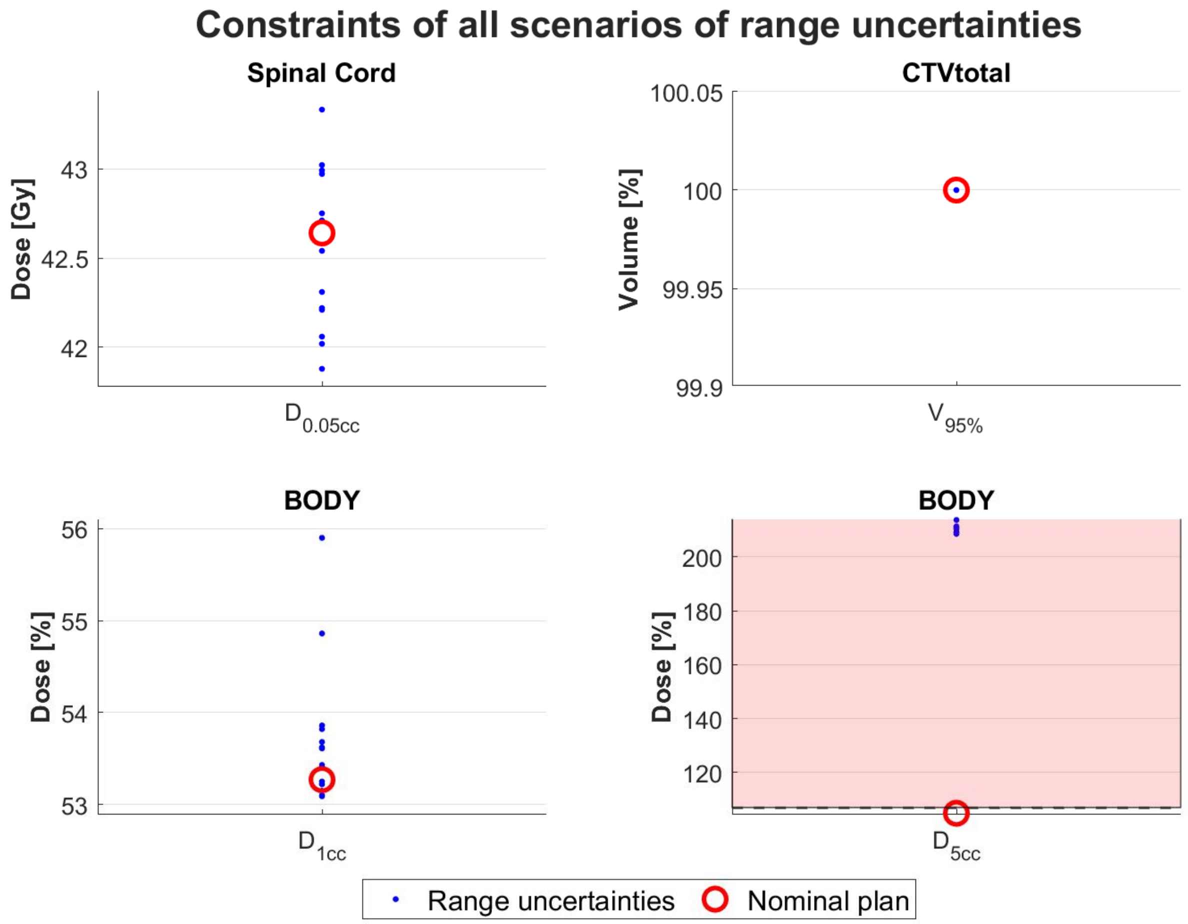

Evaluations of dose constraints for PT plan:

First priority constraints of the nominal PT plan:

Structure	Indicator	Value	Constraint
iCTVtotal	Dmean	✓ 100.9127	98% < x < 102%
iCTVtotal	V95%	✓ 100.0000	> 99%
Spinal_Cord	D0.05cc	✓ 41.6758	< 45Gy
Spinal_Cord_PRV	D0.05cc	✓ 44.1957	< 50Gy
Lungs	MLD	✓ 5.9600	< 20Gy
Lungs	V20Gy	✓ 10.8300	< 35%
Lungs	V5Gy	✓ 25.5700	< 70%
BODY	D0.05cc	✓ 104.3650	< 110%
BODY	D1cc	✓ 104.3643	< 107%

Second priority constraints of the nominal PT plan:

Structure	Indicator	Value	Constraint
iCTVtotal	Dmean	✓ 100.9127	99% < x < 101%
Lungs	V5Gy	✓ 25.5700	< 60%
Liver	V30Gy	✓ 13.3300	< 33%
Heart	MHD	✓ 14.4200	< 26Gy
Heart	V40Gy	✓ 13.5300	< 30%
Heart	V25Gy	✓ 23.9400	< 50%
Kidneys	MKD	✓ 2.1900	< 10Gy
Kidneys	V20Gy	✓ 1.6000	< 32%
Kidney_R	V6Gy	✓ 0	< 30%
Kidney_L	V6Gy	✓ 16.4200	< 30%
Bowel_Cavity	V30Gy	✓ 5.0200	< 600cc
Bowel_Cavity	V45Gy	✓ 1.1100	< 300cc
Stomach - iCTV	D0.5cc	! 103.5134	< 103%
Spleen	MSD	✓ 15.1100	< 20Gy

Constraints of all PT scenarios for robust evaluation:

Structure	Indicator	Constraint	Comment
Spinal_Cord	D0.05cc	< 50Gy	✓
BODY	D1cc	< 110%	✓
BODY	D5cc	< 107%	! 108.92Gy for i_00, 107.54Gy for i_10, 108.73Gy for i_90
CTVtotal	V95%	> 97%	✓

Constraints of all PT scenarios for range uncertainties:

Structure	Indicator	Constraint	Comment
Spinal_Cord	D0.05cc	< 50Gy	✓
BODY	D1cc	< 110%	✓
BODY	D5cc	< 107%	! 210.71% for Es_U1, 209.13% for Es_U10, 211.31% for Es_U11, 209.32% for Es_U12,...
CTVtotal	V95%	> 97%	✓

Plot of constraints of all PT scenarios for robust evaluation:

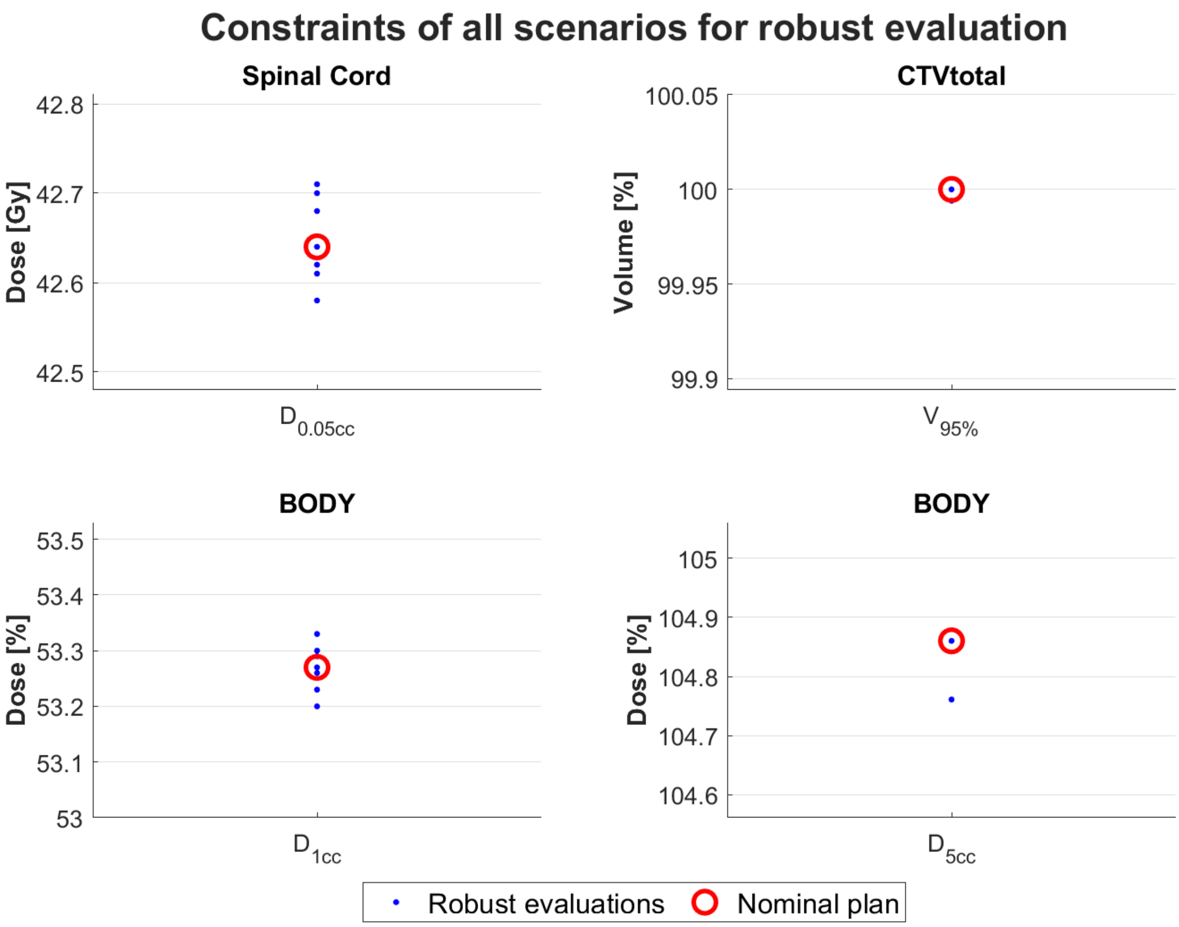

Plot of constraints of all PT scenarios for range uncertainties:

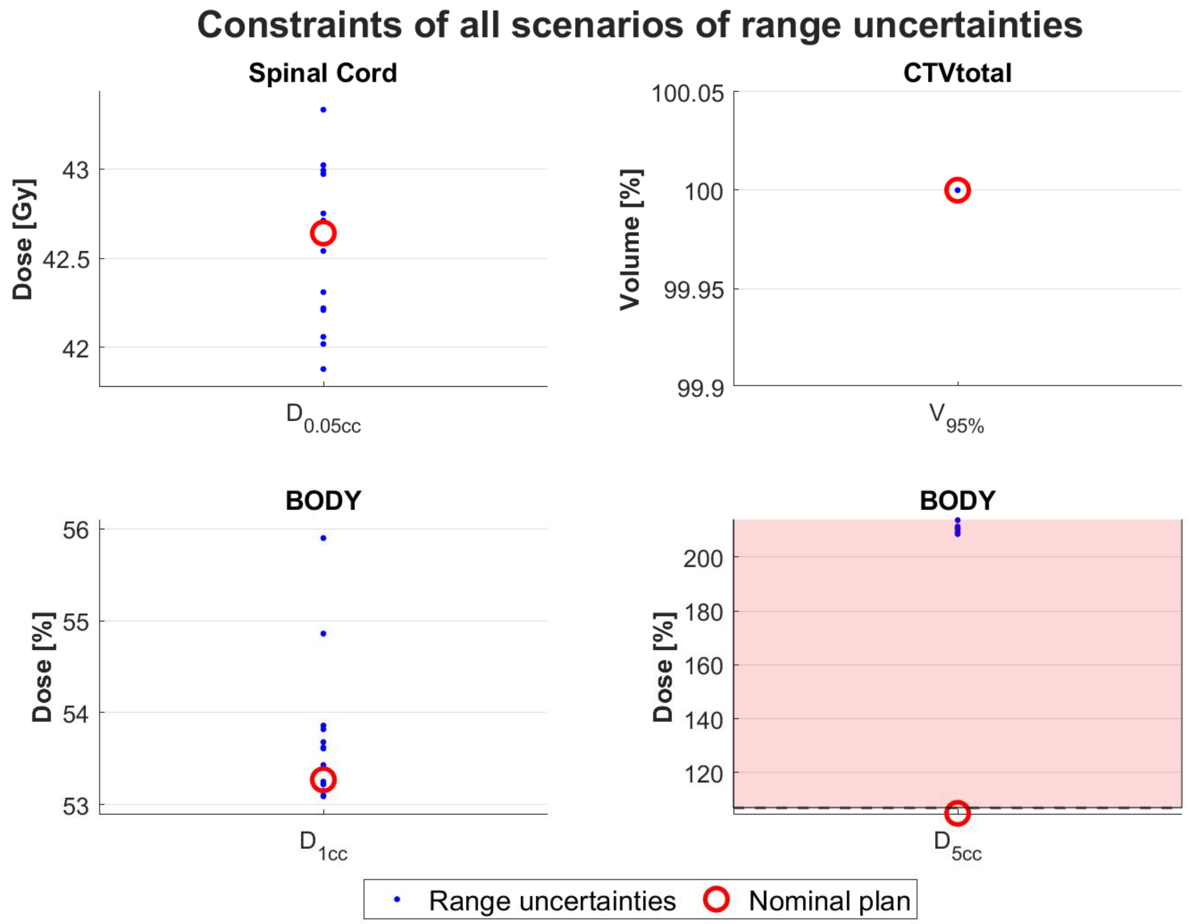

Notes:

The following can cause major deviations (re-do plan):

- Primary constraints
- Robust target coverage
- Spinal cord dose
- Robust evaluation (both setup, range and respiration)
- Proton plans: field angles OK?
- Total lung included in scan?
- XT: Scan has to be made on mid-vent CT, mid-position CT or average-CT of the planning 4D-CT scan. PT: plan is made on average-CT of the planning 4D-CT scan.

Minor deviations (think about this for the next plan):

- Robust body max dose
- Secondary constraints

Checklist for site visits

in the

PROTECT trial

Proton therapy - PT

Answer all questions. If checkboxes are present, mark these. If not, please insert text.

Proton therapy facility:

RTQA Manager:

RTQA Oncologist:

RTQA Physicist:

Date of visit:

Scanning procedures:

Maximum slice separation 2 mm ☐ Yes ☐ No

Minimum 8 phases of the 4D-CT scans are reconstructed ☐ Yes ☐ No

Which CT-calibration curve is used?

Scan length

- The scan length should include the whole target, the full extension of the lungs, and ***if target extends below the diaphragm,*** the full extension of the liver and kidneys.
- ***If target is at the level of the kidneys***, the scan should include the full extension of the kidneys.
- ***If one 4D-CT cannot encompass the full length*** as described above, two 4D-CTs should be acquired and combined into one 4D-CT encompassing the full length.

Patient positioned in the immobilization device used for treatment? ☐ Yes ☐ No

☐ Standard device ☐ Individual device

Is the same immobilization device used for the FDG-PET scan? ☐ Yes ☐ No

Is the scan acquired within three weeks prior to the start of PT? ☐ Yes ☐ No

☐ Single energy CT ☐ Dual energy CT.

A 4D-CT without contrast is used for planning and supplemented by additional 4D-CT or 3D-CT with contrast ☐ Yes ☐ No

If contrast is used at the 4D-CT, HU override is applied for PT planning ☐ Yes ☐ No

Which contrast agent is used?

How is contrast administered?

Is the patient given any information prior to scanning procedures on how to e.g. breathe?

☐ Yes ☐ No

If Yes - Please describe:

Delineations

Which image is used for target and OAR delineations?

☐ The image of the mid-ventilation phase,

☐ the image of the mid-position phase, *or*

☐ the average image of the planning 4D-CT scan

Are delineations discussed with a radiologist?

☐ Yes ☐ No

Are delineations discussed with a nuclear radiologist?

☐ Yes ☐ No

How is the FDG-PET signal used?

Are all scans co-registered? ☐ Yes ☐ No

Who performs target delineations?

Are target delineations approved? ☐ Yes ☐ No

If Yes - Who approves them?

Is the RO who made the PROTECT benchmark delineations involved in target delineation/approval?

☐ Yes ☐ No

Are fiducial markers used? ☐ Yes ☐ No

Who performs OAR delineations?

Do you use AI/atlas-based auto-segmentation of the OAR? ☐ Yes ☐ No

Are the OAR delineations approved? ☐ Yes ☐ No

If Yes – who approves them?

How is the iCTV generated?

Is the target discussed on an MDT (multidisciplinary team conference)? ☐ Yes ☐ No

If yes, who attends the MDT?

How is information on tumor malignancy obtained?

Treatment Planning - Protons

Who optimizes the treatment plan? ☐ RTT ☐ Physicist ☐ RO

Is the treatment planner who made the PROTECT benchmark treatment planning cases involved in treatment planning? ☐ Yes ☐ No

Treatment plan (for proton treatment) is based on the average-CT of the planning 4D-CT scan

☐ Yes ☐ No

For the patients randomized for proton therapy: Which phase is the photon dose plan based on?

☐ the mid-ventilation phase ☐ the mid-position phase ☐ the average-CT of the planning 4D-CT scan

How are couch/support structures taken into account?

Dose prescription ☐ 41.4 Gy/23 Fx ☐ 50.4 Gy/28 Fx

Advanced dose calculation algorithm and software

Which?

Maximum calculation grid size is $3x3x3mm^3$ ☐ Yes ☐ No

Planning technique ☐ SFUD ☐ IMPT

Number of beams?

Beam directions?

Setup errors

Which setup errors are used?

How are the setup errors determined? ☐ Published data ☐ Population based ☐ Other (please describe)

Which range uncertainty is used?

How is the range uncertainty determined? ☐ Published data ☐ Population based ☐ Other (please describe)

4D evaluation

How is 4D evaluation performed?

Procedures during treatment (online procedure)

Who is present at the first setup of the patient? (Check more than one box if relevant)

☐ RTT ☐ Physicist ☐ RO

Who is present at the remaining fractions? (Check more than one box if relevant)

☐ RTT ☐ Physicist ☐ RO

How is daily setup controlled? ☐ offline ☐ online

Who performs the online evaluation during daily match?

☐ RTT ☐ Physicist ☐ RO

How many degrees of freedom are used for the match? ☐ 3D ☐ 4D ☐ 6D

Which action is taken in case of deviations larger than accepted are observed?

☐ Call doctor. Describe action

☐ Action on deviations noted on treatment chart

☐ Other. Describe

Do you note deviations on a treatment chart? ☐ Yes ☐ No ☐ Other

Briefly, describe your online match procedure before daily treatment:

Which type of match? ☐ Bone ☐ Soft tissue ☐ Other (specify)

What are your match tolerances?

Describe actions in case of non-acceptable deviations at the daily treatment:

Which imaging modality do you use?

Describe your procedure at a breakdown/maintenance of the machine:

Please be prepared to describe the treatment procedure more thoroughly at the site visit.

Offline adaptive therapy

Do you perform an offline evaluation after treatment delivery: ☐ Yes ☐ No

Who performs the offline evaluation? ☐ RTT ☐ Physicist ☐ RO

How often do you perform offline evaluation?

Which action is taken in case of deviations larger than accepted are observed?

☐ Call doctor. Describe action

☐ Action on deviations noted on treatment chart

☐ Other. Describe

What is to be done if the deviations are not acceptable?

Who is involved in the decision to adapt the treatment?

☐ RTT ☐ Physicist ☐ RO

Do you recalculate dose if deviations are observed? ☐ Yes ☐ No

If yes, how?

Which criteria do you have for re-planning? ☐ Dosimetric ☐ Geometric ☐ Other (specify)

Where do these criteria/tolerances originate from? (Please describe)

Plan for weekly 4D-CT

Who checks target delineations? ☐ RTT ☐ Physicist ☐ RO

How is the target and spinal cord transferred to the weekly scan?

☐ Deformably ☐ Rigidly ☐ Manually ☐ Other (specify)

Who checks the doses to target and spinal cord?

☐ RTT ☐ Physicist ☐ RO

Patient specific QA procedures

How is QA performed?

☐ Measurement of plan at accelerator ☐ Log files

When is the deadline for QA?

Which criteria are used?

Which secondary dose calculation algorithm and software is used?

Which criteria?

Is QA performed on adapted plans?

What is your procedure with regards to dosimetry audits?

☐ IBA ☐ Other (please describe):

Last audit performed on

Comments

Checklist for site visits

in the

PROTECT trial

Photon therapy - XT

Answer all questions. If checkboxes are present, mark these. If not, please insert text.

Photon therapy facility:

RTQA Manager:

RTQA Oncologist:

RTQA Physicist:

Date of visit:

Scanning procedures:

Maximum slice separation 2 mm ☐ Yes ☐ No

Minimum 8 phases of the 4D-CT scans are reconstructed ☐ Yes ☐ No

Which CT-calibration curve is used?

Scan length

- The scan length should include the whole target, the full extension of the lungs, and ***if target extends below the diaphragm,*** the full extension of the liver and kidneys.
- ***If target is at the level of the kidneys***, the scan should include the full extension of the kidneys.
- ***If one 4D-CT cannot encompass the full length*** as described above, two 4D-CTs should be acquired and combined into one 4D-CT encompassing the full length.

Do you comply with these guidelines? ☐ Yes ☐ No ☐ Other (please specify)

Patient positioned in the immobilization device used for treatment? ☐ Yes ☐ No

☐ Standard device ☐ Individual device

Is the same immobilization device used for the FDG-PET scan? ☐ Yes ☐ No

Is the scan acquired within three weeks prior to the start of XT? ☐ Yes ☐ No

☐ Single energy CT ☐ Dual energy CT.

☐ 4D-CT with contrast ☐ 4D-CT combined with a 3D scan with contrast.

Which contrast agent is used?

How is contrast administered?

Is the patient given any information prior to scanning procedures on how to e.g. breathe?

☐ Yes ☐ No

If Yes - Please describe:

Delineations

Which image is used for target and OAR delineations?

☐ The image of the mid-ventilation phase,

☐ the image of the mid-position phase, *or*

☐ the average image of the planning 4D-CT scan

Are delineations discussed with a radiologist?

☐ Yes ☐ No

Are delineations discussed with a nuclear radiologist?

☐ Yes ☐ No

How is the FDG-PET signal used?

Are all scans co-registered? ☐ Yes ☐ No

Who performs target delineations?

Are target delineations approved? ☐ Yes ☐ No

If Yes - Who approves them?

Is the RO who made the PROTECT benchmark delineations involved in target delineation/approval?

☐ Yes ☐ No

Are fiducial markers used? ☐ Yes ☐ No

Who performs OAR delineations?

Do you use AI/atlas-based auto-segmentation of the OAR? ☐ Yes ☐ No

Are the OAR delineations approved? ☐ Yes ☐ No

If Yes – who approves them?

How is the iCTV generated?

Is the target discussed on an MDT (multidisciplinary team conference)? ☐ Yes ☐ No

If yes, who attends the MDT?

How is information on tumor malignancy obtained?

Treatment Planning - Photons

Who optimizes the treatment plan? ☐ RTT ☐ Physicist ☐ RO

Is the treatment planner who made the PROTECT benchmark treatment planning cases involved in treatment planning? ☐ Yes ☐ No

Treatment plan (for photon treatment) is based on

☐ the mid-ventilation phase ☐ the mid-position phase ☐ the average-CT of the planning 4D-CT scan

For the patients randomized for proton therapy: Which phase is the photon dose plan based on?

☐ the mid-ventilation phase ☐ the mid-position phase ☐ the average-CT of the planning 4D-CT scan

How are couch/support structures taken into account?

Dose prescription ☐ 41.4 Gy/23 Fx ☐ 50.4 Gy/28 Fx

Advanced dose calculation algorithm and software

Which? ____

Maximum calculation grid size is 3x3x3mm^3 ☐ Yes ☐ No

Planning technique ☐ IMRT ☐ VMAT

Do you use a standard field configuration? ☐ Yes ☐ No

Please describe:

Are non-coplanar fields used? ☐ Yes ☐ No

What is your PTV margin:

How is the PTV margin determined? ☐ Published data ☐ Population based ☐ Other (please describe)

What are your setup errors for robust evaluation?

How are the setup errors determined? ☐ Published data ☐ Population based ☐ Other (please describe)

4D evaluation

How is 4D evaluation performed?

Procedures during treatment (online procedure)

Who is present at the first setup of the patient? (Check more than one box if relevant)

☐ RTT ☐ Physicist ☐ RO

Who is present at the remaining fractions? (Check more than one box if relevant)

☐ RTT ☐ Physicist ☐ RO

How is daily setup controlled? ☐ offline ☐ online

Who performs the online evaluation during daily match?

☐ RTT ☐ Physicist ☐ RO

How many degrees of freedom are used for the match? ☐ 3D ☐ 4D ☐ 6D

Which action is taken in case of deviations larger than accepted are observed?

☐ Call doctor. Describe action

☐ Action on deviations noted on treatment chart

☐ Other. Describe

Do you note deviations on a treatment chart? ☐ Yes ☐ No ☐ Other

Briefly, describe your online match procedure before daily treatment:

Which type of match? ☐ Bone ☐ Soft tissue ☐ Other (specify)

What are your match tolerances?

Describe actions in case of non-acceptable deviations at the daily treatment:

Which imaging modality do you use?

Describe your procedure at a breakdown/maintenance of the machine:

Please be prepared to describe the treatment procedure more thoroughly at the site visit.

Offline adaptive therapy

Do you perform an offline evaluation after treatment delivery: ☐ Yes ☐ No

Who performs the offline evaluation? ☐ RTT ☐ Physicist ☐ RO

How often do you perform offline evaluation?

Which action is taken in case of deviations larger than accepted are observed?

☐ Call doctor. Describe action

☐ Action on deviations noted on treatment chart

☐ Other. Describe

What is to be done if the deviations are not acceptable?

Who is involved in the decision to adapt the treatment?

☐ RTT ☐ Physicist ☐ RO

Do you recalculate dose if deviations are observed? ☐ Yes ☐ No

If yes, how?

Which criteria do you have for re-planning? ☐ Dosimetric ☐ Geometric ☐ Other (specify)

Where do these criteria/tolerances originate from? (Please describe)

Plan for weekly 4D-CT

Who checks target delineations? ☐ RTT ☐ Physicist ☐ RO

How is the target and spinal cord transferred to the weekly scan?

☐ Deformably ☐ Rigidly ☐ Manually ☐ Other (specify)

Who checks the doses to target and spinal cord?

☐ RTT ☐ Physicist ☐ RO

Patient specific QA procedures

How is QA performed?

☐ Measurement of plan at accelerator ☐ Log files

When is the deadline for QA?

Which criteria are used?

Which secondary dose calculation algorithm and software is used?

Which criteria?

Is QA performed on adapted plans?

What is your procedure with regards to dosimetry audits?

☐ IBA ☐ Other (please describe):

Last audit performed on

Comments

PROTECT study

Site Visit Report

QA team:

XT	Comments	Remarks
Scanning		
Delineation		
Treatment planning		
Online procedure		
Offline adaptive procedure		
Weekly 4DCT		
Patient QA		
General comments		

PT	Comments	Remarks
Scanning		
Delineation		
Treatment planning		
Online procedure		
Offline adaptive procedure		
Weekly 4DCT		
Patient QA		
General comments		
